# Host-specific fluorescence dynamics in legume-rhizobium symbiosis during nodulation

**DOI:** 10.1128/aem.02154-25

**Published:** 2026-01-16

**Authors:** Chandan K. Gautam, Gayathri Senanayake, Amanda B. Pease, Mohamed A. Salem, Ahmed H. Rabia, Birgit M. Prüß, Barney A. Geddes

**Affiliations:** 1 Department of Microbiological Sciences, North Dakota State University 3323, Fargo, North Dakota, USA; 2 Department of Agricultural and Biosystems Engineering, North Dakota State University 3323, Fargo, North Dakota, USA; The University of Tennessee Knoxville, Knoxville, Tennessee, USA

**Keywords:** rhizobia, legume, fluorescence bioreporters, nodulation, deep learning

## Abstract

The legume-rhizobium symbiosis is a cornerstone of sustainable agriculture due to its ability to facilitate biological nitrogen fixation. Still, real-time visualization and quantification of this interaction remain technically challenging, especially across different host backgrounds. In this study, we systematically evaluate the efficacy of the nitrogenase system *nifH* promoter (P*nifH*) in driving expression of distinct fluorescent reporters; superfolder yellow fluorescent protein (sfYFP), superfolder cyan fluorescent protein (sfCFP), and various red fluorescent proteins (RFPs) within root nodules of determinate (*Lotus japonicus-Mesorhizobium japonicum*) and indeterminate (*Pisum sativum-Rhizobium leguminosarum*) systems. We show that P*nifH*-driven sfYFP and sfCFP yield strong, uniform, and reproducible fluorescence in nodules of both systems, facilitating reliable quantification of nodulation traits and strain occupancy. In contrast, RFPs including monomeric (mScarlet-I, mRFP1, mARs1) and multimeric (AzamiRed1.0) variants exhibited weak or inconsistent signals in pea. Notably, fluorescent labeling did not impair rhizobial competitiveness for root nodule occupancy, and P*nifH*-driven sfYFP and sfCFP reporters enabled robust multiplexed imaging in single-root and split-root assays. In the lotus, mScarlet-I worked robustly and facilitated a tripartite strain labeling system. Complementing our molecular toolkit, we established a deep learning-based analytical pipeline for high-throughput, automated quantification of nodulation traits, validated against standard ImageJ analysis. Altogether, our results identify P*nifH*-driven sfYFP and sfCFP as robust, broadly applicable reporters for legume-rhizobium symbiosis studies, while highlighting the need for optimized red fluorophores in some contexts. The integration of validated promoter-reporter constructs with state-of-the-art computational approaches provides a scalable framework for dissecting the spatial and competitive dynamics of plant-microbe mutualisms.

**IMPORTANCE:**

The legume-rhizobium symbiosis is central to sustainable agriculture through its capacity for biological nitrogen fixation, yet tools for real-time, quantitative visualization of this interaction remain limited. Here, we demonstrate that the *nifH* promoter (P*nifH*) effectively drives expression of superfolder yellow (sfYFP) and cyan (sfCFP) fluorescent proteins in both determinate (*Lotus japonicus-Mesorhizobium japonicum*) and indeterminate (*Pisum sativum-Rhizobium leguminosarum*) nodules. These reporters enable robust, reproducible fluorescence without impairing rhizobial competitiveness, supporting multiplexed imaging and quantitative nodulation analyses. By contrast, red fluorescent proteins exhibited host-dependent variability, underscoring the need for improved red fluorophores. Integration of validated promoter-reporter constructs with a deep learning-based image analysis pipeline establishes a scalable framework for high-throughput assessment of nodule occupancy and symbiotic dynamics. This work provides a practical molecular and computational toolkit for dissecting plant-microbe mutualisms across diverse host systems.

## INTRODUCTION

The symbiotic interaction between legumes and rhizobia is governed by complex genetic and molecular mechanisms (1). Advances in molecular biology and genomics have significantly deepened our understanding of these symbiotic events. A pivotal breakthrough was the identification of three main groups of rhizobial genes (*nod, nif,* and *fix*), which revolutionized research on nodulation from the bacterial perspective (2–6). Furthermore, with the advent of transcriptomics in nodulating legumes, researchers have been able to achieve an even broader view of the molecular events taking place during legume-rhizobium symbiosis (7, 8). Legumes produce root exudates which include flavonoids and iso-flavonoids (9–11). These compounds trigger the biosynthesis of rhizobial Nod factors (lipo-chitooligosaccharide) (12). These products of the *nod* genes trigger initial plant responses such as root hair curling and cortical cell division upon the contact with the rhizobia, thus initiating infection and nodule formation (7, 13). After successful infection, *nif* genes encode the nitrogenase enzyme complex, primarily dinitrogenase reductase, which is essential for converting atmospheric nitrogen (N_2_) to ammonium (NH_4_^+^) (14). The *fix* genes code for metabolic proteins required for nitrogenase function, which are regulated under the low-oxygen (microaerobic) conditions present within the nodule (15, 16).

Early studies faced significant gaps in knowledge regarding the spatial distribution and real-time monitoring of rhizobia within the legume host. This was primarily due to limitations in technology; traditional approaches, such as staining and light microscopy, could only provide static snapshots (17, 18). While the advent of gene-specific PCR fingerprinting, strain-specific nucleotide barcodes employing next-generation sequencing and metagenomic techniques improved resolution and enabled investigation of the effectiveness and competitiveness of multi-strain competition, their use remains constrained by complexity and cost (17, 19–23).

A breakthrough in studying real-time plant invasion dynamics by rhizobia was achieved with the development of green fluorescent protein (GFP)-based reporters in *Sinorhizobium meliloti*, driven by the constitutive tryptophan promoter, *trp* (P*trp*) from *Salmonella typhimurium*, and the successful visualization of this reporter in the host *Medicago sativa* (alfalfa) (24). In parallel, utilizing the *nifH* nitrogenase promoter to drive the expression of the chromogenic beta-glucuronidase marker gene *gusA* within transposons introduced to *Rhizobium tropici* CIAT899, demonstrated a reliable reporter of nitrogen fixation in *Phaseolus vulgaris L. cv. Riz 30* (common bean) (25). This system was useful for comparing the competitiveness of labeled strains to unlabeled isogenic parents. The system was later improved to allow concurrent labeling of two different strains by the inclusion of an alternate thermostable beta-galactosidase-encoding gene, *celB*, and implemented in the *R. leguminosarum-Pisum sativum* system (26). Although these studies utilized the *nifH* promoter system (specific to nitrogen fixation), the staining procedure for chromogenic markers terminated cellular activity, preventing live imaging. In later studies, *S. typhimurium* P*trp*-driven DsRed and GFP were introduced into different strains of *S. meliloti* to study competitiveness (17, 27). However, a limitation with this reporter system was its non-specificity in reporting nodulation events. Nevertheless, this research laid the foundation for further improvement of reporter systems, which can be used explicitly to study rhizobial dynamics during nodulation within the host. Currently, *nifH* promoter (P*nifH*)-driven fluorescent reporters are the gold standard for monitoring effective nodulation and nitrogen fixation in legume-rhizobium studies (22, 25, 28, 29). The *nifH* gene encodes a key nitrogenase component and is specifically activated under the microaerobic conditions inside nodules where nitrogenase functions, making it a precise marker of active nitrogen-fixing symbiosis (25, 30, 31). Its expression is regulated by the FixJ/FixL system via FixK and NifA intermediates which are functional under microaerobic conditions, reflecting both nitrogen fixation gene activation and the low-oxygen nodule environment (3, 31–33). This specificity enables the accurate quantification of functional nodules that contribute to plant nitrogen nutrition. In contrast, *nod* gene promoters (e.g., *nodABC*) are activated early in the legume-rhizobium interaction, leading to Nod factor synthesis, which triggers nodule initiation (3, 30, 34). Their activity typically decreases sharply after infection thread formation and the onset of nodule organogenesis (34). Thus, the activity of *nod* promoters does not reliably reflect the presence of mature, functioning nodules, and their use would result in false positives unrelated to effective nitrogen fixation. The *fix* gene promoters exhibit strong regulation by oxygen levels and can be expressed even in free-living rhizobia when exposed to low-oxygen conditions outside the plant (32, 33). Therefore, the expression from these promoters is not strictly limited to symbiotic conditions, resulting in reduced specificity if used as a marker for nodulation events.

In this study, we systematically evaluated the efficacy of a P*nifH*-driven fluorescent reporter system within legume-rhizobium symbiosis by employing three spectrally distinct fluorescent proteins-superfolder yellow fluorescent protein (sfYFP), superfolder cyan fluorescent protein (sfCFP), and red fluorescent proteins (RFPs: mScarlet-I, AzamiRed1.0, mRFP1, and mARs1)—each expressed under the control of the P*nifH* promoter (35–39). These constructs were tested in two model legume-rhizobium associations: *Pisum sativum* (pea)*-Rhizobium* (indeterminate nodules) and *Lotus japonicus* (lotus)*-Mesorhizobium* (determinate nodules) (40). Determinate nodules are globular structures that cease growth shortly after initiation due to the absence of a persistent meristem, as observed in soybean and lotus (40, 41). Conversely, indeterminate nodules retain an active apical meristem, resulting in an elongated, zonated structure characteristic of pea, alfalfa, and clover (40, 41). Our analyses revealed that sfCFP and sfYFP consistently produced robust and uniform fluorescence signals in nodules of both pea and lotus. To further benchmark reporter performance, we compared these results to those obtained using a constitutive σ−70 consensus promoter, thereby assessing the impact of promoter choice on fluorescence intensity and spatial distribution. To complement these molecular tools with scalable phenotyping, we built a deep learning (DL) workflow that converts fluorescence micrographs into quantitative readouts of symbiotic performance. By integrating spectral discrimination with high-throughput, quantitative phenotyping, our approach not only advances the toolkit for studying legume-rhizobium interactions but also opens new avenues for dissecting the spatial and competitive dynamics of symbiotic partnerships. This scalable framework has the potential to transform how we interrogate plant-microbe mutualisms, enabling deeper, system-level insights into the factors that govern symbiotic efficiency and resilience.

## MATERIALS AND METHODS

### Bacterial strains and growth conditions

Plasmid constructs carrying promoter-driven fluorescent protein-encoding genes were assembled in *Escherichia coli*. Cultures of *E. coli* were maintained at 37 °C in Luria-Bertani (LB) medium (42) supplemented with appropriate antibiotics (Table S1). Construct assembly was carried out using a BsaI-based Golden Gate cloning strategy, incorporating either the P*nifH* promoter or synthetic universal promoters (P*uni*). The P*uni* included the *E. coli* constitutive σ−70 consensus J23106 (promoter) and B0032m (ribosome binding site) from the CIDAR toolkit (43), which was originally derived from the Anderson promoter library. Plasmid components, including the fluorescent protein sequences, were obtained from Addgene (https://www.Addgene.org/) or synthesized by commercial providers and subsequently assembled in-house. All constructs were sequence-verified in *E. coli* before mobilization into rhizobial recipient strains via conjugation. The rhizobial strains used in this study are *R. leguminosarum* bv. *viciae* 3841 (44) (reclassified as *R. johnstonii* [45]) and *M. japonicum* strain R7A (formerly known as *M. loti*). Additionally, two novel *Rhizobium* strains, G22 and G11, isolated from North Dakota (USA), were also used in this study. The fluorescent proteins used to label rhizobia are sfYFP, sfCFP, mScarlet-I, mARs1, AzamiRed1.0 (Azami Red), and mRFP1 (35–39). The details of the plasmids and the sequence of fluorescent proteins are presented in Table S1 and S2. Both rhizobium strains were cultivated at 30°C in tryptone-yeast (TY) medium, a complex, non-selective growth medium suitable for a broad range of bacteria (46), supplemented with 2.5 mM CaCl_2_ and appropriate antibiotics Tale S1). The resulting fluorescently labeled rhizobia were used for all plant inoculation experiments.

### Design of custom synthetic *Mesorhizobium nifH* promoter

For *Rhizobium*, the synthetic consensus promoter Rlv P*nifH* described by (22) was used to drive different fluorophores ( Table S1, S2).

For *M. japonicum,* a custom synthetic *Mesorhizobium nifH* promoter was designed and implemented based on multiple sequence alignment of *nifH* upstream regions from reference strains of several symbiotic *Mesorhizobium* species. These included *M. amorpha* CCNWGS0123, *M. ciceri* bv. *biserrulae* WSM1284 and WSM1271, *M. jarvisii* ATCC33669, *M. ciceri* CC1192A, *M. muleiense* AHPC30, *M. loti* TONO, and *M. loti* MAFF303099. Consensus sequences from a region of high homology spanning 295 bp upstream of the *nifH* coding sequence were used to design the synthetic sequence (Fig. S1; Table S2).

### Seed sterilization and germination

*P. sativum* CDC Striker (47) field peas and *L. japonicus cv*. Gifu seeds were used in this study. Pea seeds were surface sterilized in 50 mL Falcon tubes using sequential treatments: 95% ethanol (30 s) followed by 2% sodium hypochlorite (5 min), with 10 sterile ddH₂O rinses. Sterilized seeds were germinated on 0.7% (wt/vol) agar plates in the dark at room temperature for 48 h. Lotus seeds were scarified with concentrated sulfuric acid (15 mL, 10–12 min), rinsed 10 times with sterile ddH₂O, and then sterilized in 2.5% sodium hypochlorite (2 min) followed by 10 washes with sterile ddH₂O. After soaking in ddH₂O for 4 h, seeds were germinated on 0.8% (wt/vol) water agar plates wrapped in foil and incubated in darkness at room temperature for 48h.

### Plant growth and inoculation

The germinated seedlings of pea and lotus were cultivated in Leonard jars filled with a 1:1 (wt/wt) mixture of quartz sand and vermiculite. Each jar was supplemented with 250 mL of sterile nitrogen-free rooting solution (Table S3) (48) for pea or with Jensen’s medium supplemented with 0.5 mM KNO₃ for lotus (Table S4) (49). The Leonard jars containing the quartz-vermiculite substrate and supplemented nutrient medium were autoclaved and then allowed to cool to room temperature before the germinated seedlings were transferred. Plants were maintained in a controlled growth chamber under long-day conditions. For pea, conditions consisted of an 18 h light/6 h dark photoperiod with day/night temperatures of 21°C/17 °C. Lotus was grown under a 16 h light/8 h dark cycle at a constant temperature of 20°C.

Three days after sowing, seedlings were inoculated with 10 mL of the respective rhizobial suspension expressing fluorescent proteins under either the P*nifH* or P*uni* promoter. Pea seedlings were inoculated with *R. leguminosarum* (OD₆₀₀ = 0.06), while lotus seedlings received *M. japonicum* (OD₆₀₀ = 0.08) inoculum.

The co-inoculation experiments were performed independently for *Rhizobium,* and *Mesorhizobium* on their respective hosts, pea and lotus. Equal volumes of the respective rhizobial cultures (with similar OD_600_ values) with unique fluorescent labels were mixed to yield a total volume of 10 mL, which was then applied to the seedlings in a Leonard jar. The final OD of the inoculum was the same as above, *Rhizobium* (OD₆₀₀ = 0.06) and *Mesorhizobium* (OD₆₀₀ = 0.08).

### Split-root system preparation

For split-root experiments, pea seeds were surface-sterilized and germinated on sterile 0.7% agar plates, as described above. The germinated seedlings were excised at the junction of the hypocotyl and radicle using a sharp sterile blade, inside a laminar hood. The seedlings were then carefully transferred into the holes of a 1 mL pipette tip box filled with sterile nitrogen-free ½-strength Hoagland nutrient solution (50) (Table S5), ensuring that the cut region remained in contact with the nutrient solution to prevent dehydration (Fig. S2). The lid of the tip box was loosely placed on top, and the setup was maintained in a growth chamber under a photoperiod of 18 h light at 21°C and 6 h dark at 17°C for 4–5 days to allow the formation of adventitious roots.

After this period, all but two morphologically similar lateral roots were carefully removed using precision scissors inside the laminar hood. The remaining two roots were positioned such that each extended into a separate hole of the same tip box, allowing for individual development. The lid was kept loosely closed to avoid shoot constriction and minimize damage from condensed water. After another 12–14 days of growth, plants with clearly separated elongated-root systems were removed, and the split-roots were transplanted into separate Leonard jars (placed side by side, as shown in Fig. S2) containing a 1:1 (wt/wt) mixture of quartz sand and vermiculite, along with 250 mL of sterilized nitrogen-free rooting solution (Table S3; Fig. S2) (48). The plants were kept in a growth chamber for hardening for 2 days before being subjected to various treatments. The complete workflow is illustrated in Fig. S2.

### Sample harvesting and imaging

Pea roots (including split-roots) were harvested 3 weeks post-inoculation (*wpi*), while lotus roots were collected five *wpi*. After carefully washing the samples in tap water, whole plants were stored at 4°C with roots wrapped in damp paper towels until imaging. Prior to imaging, roots were trimmed to remove non-nodulated regions and arranged on a single-well cell culture plate (pea) or a microscope slide (lotus).

For pea, fluorescence microscopy was conducted using a *Biotek Cytation 5* (version 3.10) equipped with a 1.25× PL APO objective, while for lotus, 4× PL APO was used. Image acquisition settings for Bright Field (BF) and fluorescence channels, with excitation/emission wavelengths optimized for YFP (500, 542 nm), RFP (530, 593 nm), and CFP (445, 510 nm), were tailored for each bioreporter. For pea nodules expressing fluorescent reporters under either the P*nifH* or P*uni* promoters, the imaging parameters are provided in Table S6. While, for lotus nodules, the imaging parameters are mentioned in Table S7. Focus was set either using autofocus or fixed at a height of 1,000 μm with a bottom elevation of 0.2 μm. Since P*uni*-driven fluorophores do not require a nodule-like environment to be functional, we used these settings to visualize them on a TY agar plate (Fig. S3A and B).

### Nodule counting and quantification of nodulation parameters

The images obtained from the *Cytation 5* were subjected to visual quantification of fluorescent and non-fluorescent nodules, as well as ImageJ-based quantification.

Fluorescent nodule number and intensity quantification using ImageJ was done as per the description below:

The key parameters used were minimum/maximum intensity, brightness adjustment, hue, saturation, brightness thresholds, and particle size filter. For each sample, individual fluorescence channels were adjusted to optimize visibility of nodules by modifying the minimum and maximum intensity and overall brightness. Color thresholding was subsequently applied using hue, saturation, and brightness parameters tailored to each fluorescent reporter. Automated particle analysis was then used to determine nodule number and extract fluorescence intensity values, with particle-size filters set to exclude background noise and non-nodular structures. Because closely clustered nodules may occasionally be merged during automated segmentation, manual-assisted correction was performed to ensure accurate quantification. The “Cell Counter” plugin was used to validate and adjust automated counts, particularly in regions with highly concentrated fluorescence. Nodules were examined at higher magnification, and counts were corrected as needed. Incorrectly marked objects could be removed, and updated results were generated directly through the plugin interface. Together, these automated and assisted approaches ensured robust quantification of nodule number and fluorescence intensity, even in cases involving clumped nodules.

### Data visualization and statistical analyses

All statistical analyses of nodule count data were performed using the built-in analysis tools of GraphPad Prism (version 10.5.0). Depending on the experimental design, either one-way or two-way ANOVA was applied. Post-hoc comparisons were carried out using Tukey’s Honestly Significant Difference (TukeyHSD) test, and results were summarized using a compact letter display to represent all pairwise comparisons. Data visualization, including all plots and graphs, was also generated using GraphPad Prism (version 10.5.0).

### Overview of imaging and computational workflow

**Fig 1:**
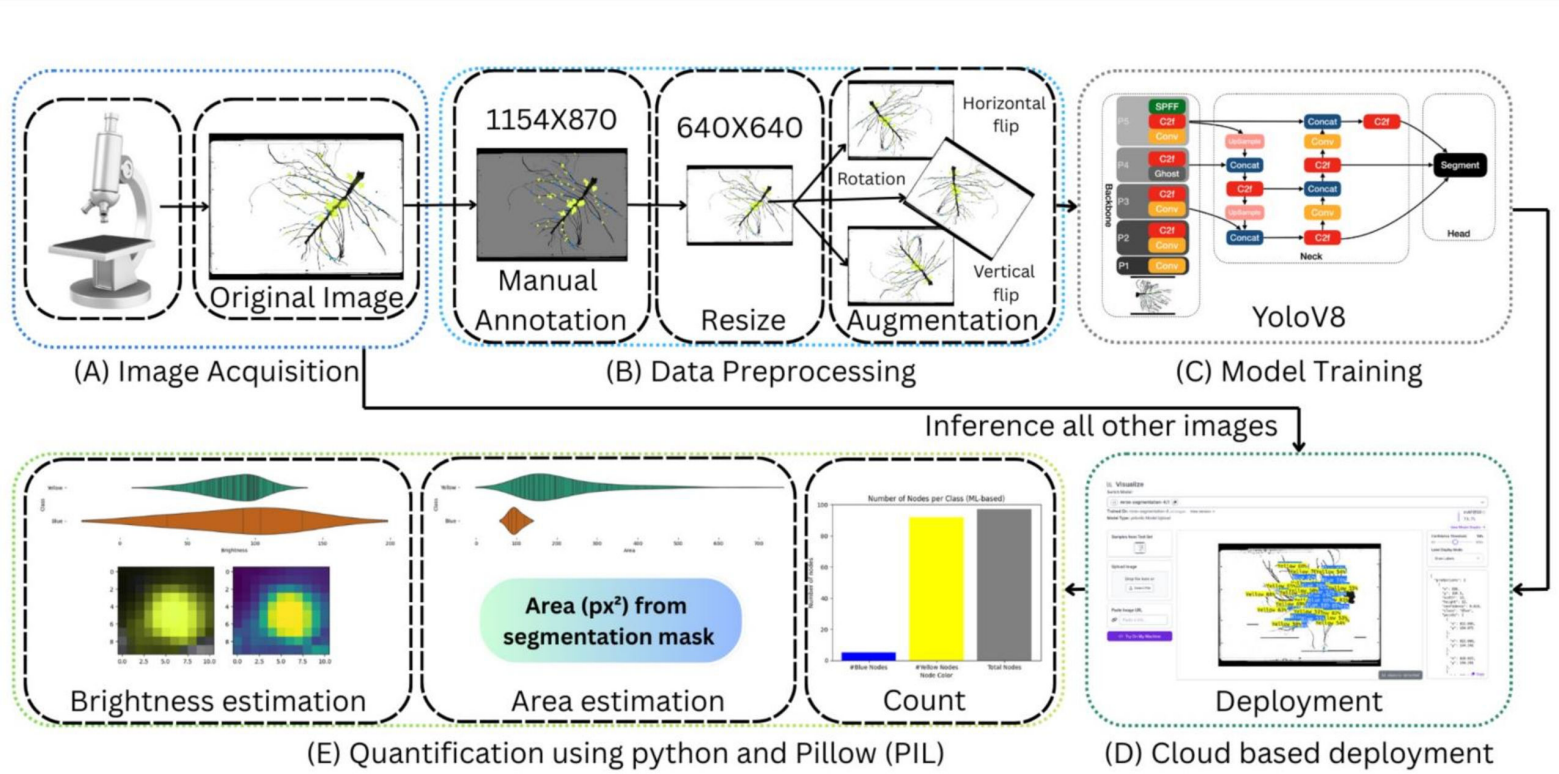
High-level workflow for imaging, deep-learning-based segmentation, and automated quantification of fluorescent nodules. Raw microscopy images were collected and manually annotated before resizing and augmentation to create a training set. A YOLOv8-seg model was fine-tuned to detect and segment nodules by fluorophore class. After training, the model was deployed for remote inference on all remaining images. Postprocessing steps included brightness estimation, area measurement, and class-specific nodule counts using Python and PIL. Final outputs were aggregated for downstream visualization and cloud-based deployment.

To provide a consistent framework for interpreting the results, we outline here the full imaging and computational pipeline used for nodule detection, classification, and quantification (Fig. 1). The workflow consists of five conceptual stages: (i) image acquisition, (ii) preprocessing, (iii) deep-learning-based segmentation, (iv) quantitative feature extraction, and (v) model evaluation and comparison with manual counting.

#### Image acquisition

Fluorescent root images were collected under standardized illumination and magnification settings to ensure consistent detection of sfCFP-, sfYFP-, and mScarlet-I expressing nodules. These raw RGB images served as the input for both manual and automated analysis.

#### Preprocessing

Before model training or inference, all images were normalized, cropped, and resized to a uniform resolution. A subset of images was manually annotated to identify individual nodules and their reporter class, forming the basis for training, validation, and testing. Standard data-augmentation steps (rotation, flips, and contrast adjustments) were applied to increase data set diversity.

#### Deep-learning segmentation

Nodule detection was performed using an instance-segmentation model fine-tuned on the annotated data set. The model generates both class labels and pixel-wise masks for each nodule. These predictions were subsequently filtered by confidence thresholds to remove low-likelihood detections.

#### Quantitative analysis

For each segmented nodule, we extracted two categories of measurements: (i) morphometric features (projected area in pixel units converted to physical dimensions) and (ii) fluorescence intensity, computed from class-specific RGB channel averages within each segmentation mask. In addition, the spatial relationship between nodules and the primary root was assessed by computing the shortest navigable distance along the filtered root skeleton. All metrics were aggregated across images for downstream statistical analyses.

#### Model evaluation and manual comparison

A fixed subset of images was reserved for validation and testing to quantify detection performance. Automated counts were compared with manually curated ground-truth counts to evaluate model accuracy and assess where manual correction remained necessary. This framework standardized the transition from raw microscopy data to biologically interpretable metrics and helps distinguish when automation is sufficient and when human inspection remains important.

### Deep learning-based quantification of nodulation traits

To enable automated, high-throughput detection and spatial quantification of nodulation events, we developed a deep learning (DL) pipeline utilizing instance segmentation and post-inference morphometric analysis (51). This pipeline was designed to detect fluorescent nodules, classify them by reporter color, quantify their area and brightness, and evaluate their spatial relationship to the main root.

Initial training images were manually annotated using Roboflow to label fluorescent nodules according to fluorophore class: *Cyan* (sfCFP), *Yellow* (sfYFP), and *Red* (mScarlet-I and variants). A total of 16 representative images were collected, 8 of which were manually segmented and augmented to generate a 15-image training set. The remaining images were split into four for validation and four for testing to assess model performance. The YOLOv8s-seg model was then fine-tuned using these annotations (52). The model was trained for 100 epochs with the AdamW optimizer with a learning rate of 0.001429 and momentum of 0.9 to optimize detection accuracy across both model systems.

### Remote inference and postprocessing

For image analysis, we employed the Roboflow Inference SDK, querying the trained model remotely using the InferenceHTTPClient. Images were first converted to high-quality JPEGs to ensure compatibility and minimize artifacts. The inference returned class labels, bounding boxes, and confidence scores for each predicted nodule. Predictions were filtered using a confidence threshold of 0.3 to exclude spurious detections. The API endpoint used was detect.roboflow. For each detected nodule, we quantified both its projected area and its mean fluorescence brightness.

### Brightness and area calculation

To assess fluorescence intensity, we cropped each region of interest in the original RGB image according to those bounding-box coordinates. Then, we computed the per-channel mean pixel values using the Python Imaging Library (PIL). Because each reporter has a distinct emission profile, we tailored our brightness calculation accordingly: nodules expressing sfCFP (the “Blue/ Cyan” class) were characterized using only the blue-channel mean, while those expressing sfYFP or mScarlet-I (the “Yellow” and “Red” classes) were quantified by averaging their red and yellow channel means to capture their mixed-spectrum signal. By combining these morphometric and photometric measurements, we derived robust metrics of nodule size and relative reporter expression intensity, enabling direct comparisons of promoter activity and colonization efficiency across treatments. The projected area, derived directly from the model’s segmentation output, was then converted to square millimeters (mm²).

### Batch analysis and aggregation

The analysis pipeline was designed to batch-process all .png images located within the working directory, allowing for efficient evaluation of large data sets. For each image, the model predictions were parsed to compute key statistics for each nodule class, including the total number of nodules, the average and total projected area (in pixels squared), and the average and total brightness, normalized on a 0–100 scale. These metrics were then aggregated into a structured summary table using the pandas library, facilitating downstream statistical analysis and visualization. The resulting DataFrame formed the basis for the violin plots and bar charts presented in the results section, which illustrate trends in nodule size distributions, relative class frequencies, and variation in fluorescence intensity across experimental conditions.

### Root detection and distance measurement

Although the segmentation model reliably identified nodules within each image, extracting the primary root structure required classical image processing techniques. To achieve this, the images were first thresholded and subjected to morphological filtering to produce a clean binary mask of the root. The distance between each detected nodule and the primary root was then calculated using a path-based approach. This method involved three key steps: first, nodules were computationally excluded from the binary mask to isolate the root network; second, a spatial filtering step retained only those pixels located within a defined proximity to the root structure, effectively removing noise and unrelated features; and third, the shortest navigable path between each nodule and the root was computed based on the retained pixel network. This approach enabled accurate modeling of spatial relationships within the root system while avoiding the limitations of Euclidean (straight-line) distance measurements. The resulting spatial data revealed biologically meaningful patterns, including a tendency for sfCFP-tagged strains to colonize away from the main root, especially in co-inoculation experiments.

This DL-enabled workflow was essential for analyzing complex data sets, particularly in split-root co-inoculation experiments. By leveraging distinct fluorescent tags and automated classification, we quantified nodule distribution and reporter activity with high spatial and spectral resolution. The ability to batch-process images and extract both biological and signal-level metrics significantly reduced manual effort and inter-observer variability.

## RESULTS

### PnifH effectively drives reliable expression of three unique fluorophores in lotus nodules but only two in pea

**Fig 2:**
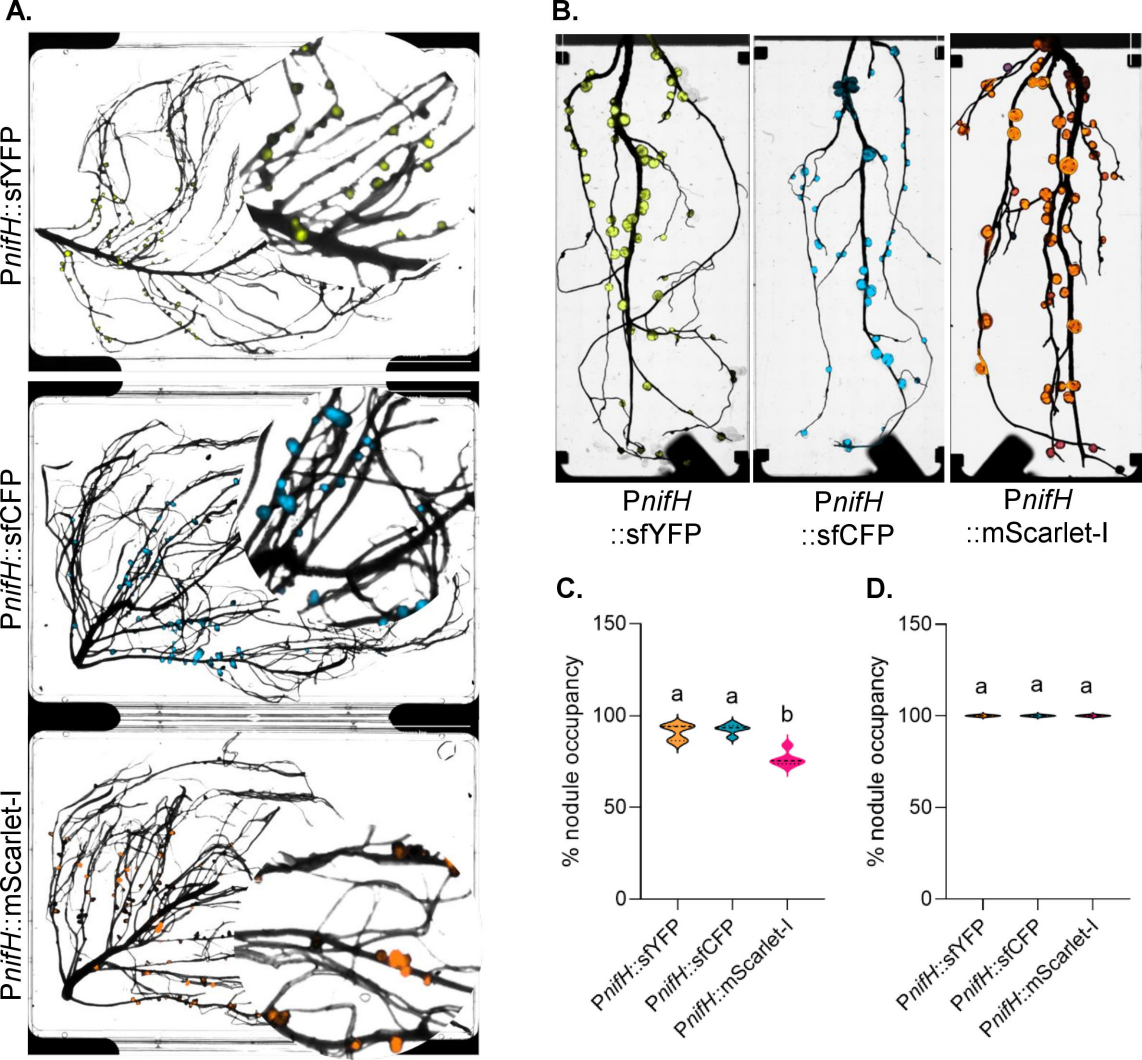
P*nifH*-driven expression of fluorescent proteins in *P. sativum* and *L. japonicus* nodules. (**A**) *Cytation 5* imaging of root nodules from *P. sativum* inoculated with *R. leguminosarum* strains expressing sfYFP, sfCFP, or mScarlet-I under the control of the P*nifH* promoter. A portion of the image has been zoomed in on a circle for better visualization. Representative images are shown from five independent biological replicates. (**B**) *Cytation 5* imaging of root nodules from *L. japonicus* inoculated with *M. japonicum* strains expressing sfYFP, sfCFP, or mScarlet-I under the control of the P*nifH* promoter. Representative images are shown from six to nine independent biological replicates. (**C**) Percentage of fluorescently occupied nodules from *P. sativum* samples shown in panel A. Data represent five independent biological replicates. (**D**) Percentage of fluorescently occupied nodules from *L. japonicus* samples shown in panel B. Data represent six to nine independent biological replicates. Distinct letters above bars indicate statistically significant differences (*P* < 0.05) as determined by one-way ANOVA followed by Tukey’s HSD test.

To develop a multiple-output reporter system that specifically marks nodule formation, accurately reflects nitrogen fixation activity, and remains unperturbed by the imposed fitness costs prior to host infection, we assembled a suite of fluorescent reporters driven by the P*nifH* promoter. This design, similar to previously described systems (22), enables reporter activity to correlate with both bacteroid abundance and the resulting nodule size. For *Rhizobium,* a synthetic consensus *Rhizobium* P*nifH* promoter was adopted from references 22. For *M. japonicum,* we designed a custom synthetic consensus *Mesorhizobium nifH* promoter based on multiple sequence alignment of *nifH* upstream regions from reference strains from several symbiotic *Mesorhizobium* species (Fig. S1; Table  S2). The promoters and fluorescent modules were combined in a medium-copy-number pBBR1 backbone that included the *par* locus, enabling stable environmental gene expression (53). These bioreporters expressed sfYFP, sfCFP, and mScarlet-I under the control of the P*nifH* promoter and were introduced to wild-type *R. johnsonii* Rlv3841 and *M. japonicum* R7A strains through conjugation. These engineered strains were used to inoculate pea and lotus seedlings. The roots of the nodulated plants were removed from pots and imaged for fluorescence (Fig. 2).

Fluorescence imaging revealed strong reporter expression in most nodules in both pea and lotus, confirming successful reporter system functionality (Fig. 2A and  B). In pea, however, the proportion of fluorescent nodules was lower across all three reporter variants (sfYFP, sfCFP, and mScarlet-I) compared to lotus (Fig. 2C and D). This reduction was most pronounced for the mScarlet-I reporter, where approximately 20% of nodules in pea exhibited weak or no detectable signal (Fig. 2A and C). Adjusting imaging parameters, including increasing gain, enhanced the fluorescent signal intensity but also resulted in elevated background noise and the appearance of pseudo-signals from root tissues. Interestingly, in lotus inoculated with mScarlet-I labeled rhizobia, nearly all nodules exhibited fluorescence. Intensity and abundance of the red fluorescence were comparable to those observed with sfCFP or sfYFP (Fig. 2B and D). Quantification of fluorescent nodules indicated a nearly consistent pattern of P*nifH*-driven expression of sfYFP and sfCFP across biological replicates in both the plant systems (Fig. 2C and D).

### Evaluation of inconsistent red fluorescent protein expression in pea nodules

In fluorescence labeling within living systems, monomeric fluorescent proteins are generally preferred due to their stability and consistent subcellular localization (39). In lotus, the monomeric RFP mScarlet-I (35), driven by synthetic P*nifH* sequences (Fig. S1B; Table  S2), performed effectively (Fig. 2B and D). However, the same fluorophore showed poor performance in pea (Fig. 2A and C). We investigated whether other monomeric RFPs could enhance performance in pea. We tested monomeric RFPs such as mRFP1 and mARs1, along with a multimeric RFP, AzamiRed1.0, driven by P*nifH* (36–39). Concurrently, to evaluate if the unreliable expression of RFPs was promoter-dependent, we compared the fluorescence of these RFPs driven by a constitutive promoter (P*uni*) (43). We used a constitutive promoter to address the possibility that nodulation or bacteroid-specific physiological constraints, such as microaerobic conditions, might limit reporter performance under the P*nifH*.

**Fig 3:**
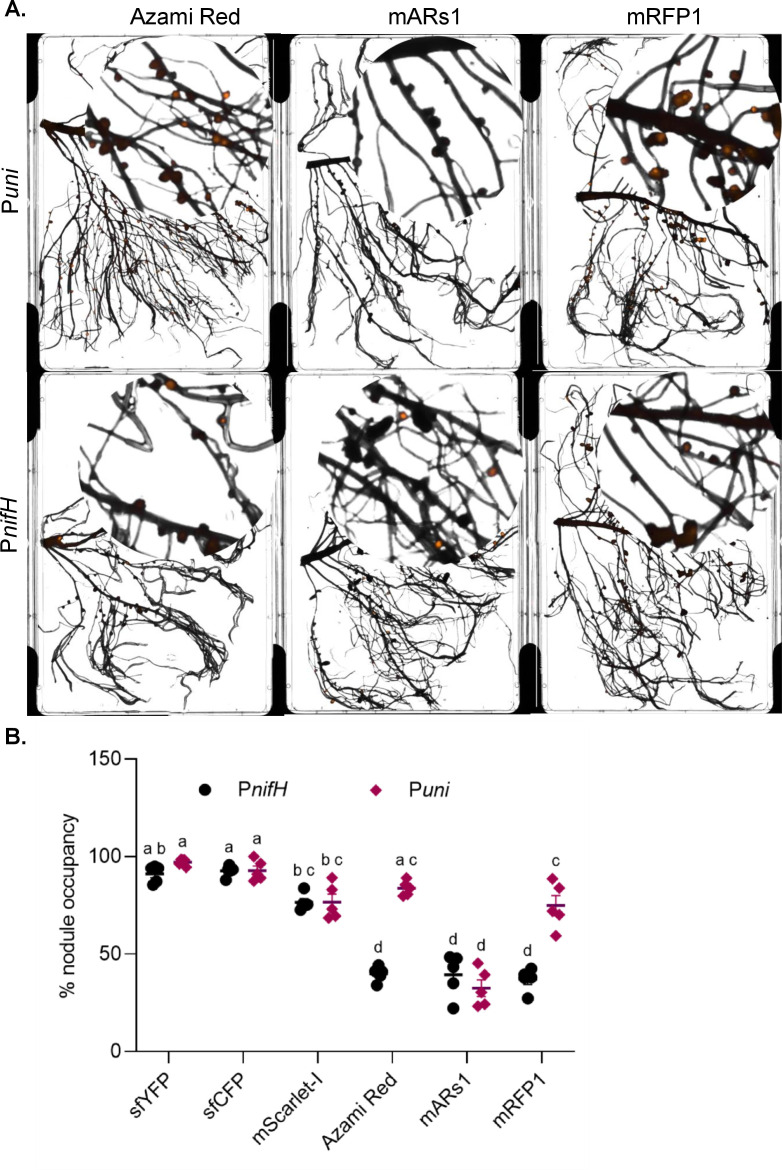
Expression of red fluorescent proteins (RFPs) in *P. sativum* nodules under P*nifH* and P*uni* promoters, and % nodule occupancy. (**A**) *Cytation 5* imaging of nodules expressing Azami Red, mARs1, or mRFP1 driven by either the P*nifH* or P*uni* promoter. A portion of the image has been zoomed in on a circle for better visualization. Representative images are shown from five independent biological replicates. (**B**) Percentage nodule occupancy of *R. leguminosarum* (with P*nifH* and P*uni* driven fluorescent labels) in *P. sativum*. Data represent five independent biological replicates. Distinct letters above bars indicate statistically significant differences (*P* < 0.05) as determined by two-way ANOVA followed by Tukey’s HSD test.

Across both the P*nifH* and P*uni* promoters, all RFP variants exhibited inconsistent and generally weak fluorescence (Fig. 3A and B; Fig. S4, S5A and B). Quantification of fluorescent nodules indicated that the use of P*uni* did not change the proportion of fluorescent nodules for mARs1 and mScarlet-I but increased significantly for Azami Red and mRFP1, compared to their respective P*nifH*-driven fluorophores (Fig. 3B); nevertheless, the overall signal intensity remained low (Fig. 3A). We also evaluated the P*uni*-based fluorescence in lotus; however, in this system, it resulted in extremely low signal intensities, requiring high-gain imaging, which, in turn, elevated background autofluorescence in the roots (Fig. S5C).

Altogether, these data indicate that in our system, P*nifH*-driven red (mScarlet-I), cyan (sfCFP), and yellow fluorophores (sfYFP) function effectively as nodule bioreporters in the lotus-*Mesorhizobium* system, while sfYFP and sfCFP proved more reliable than mScarlet-I in the pea*-Rhizobium* system.

### Fluorescent labels do not affect rhizobium effectiveness or competitiveness when co-inoculated

**Fig 4:**
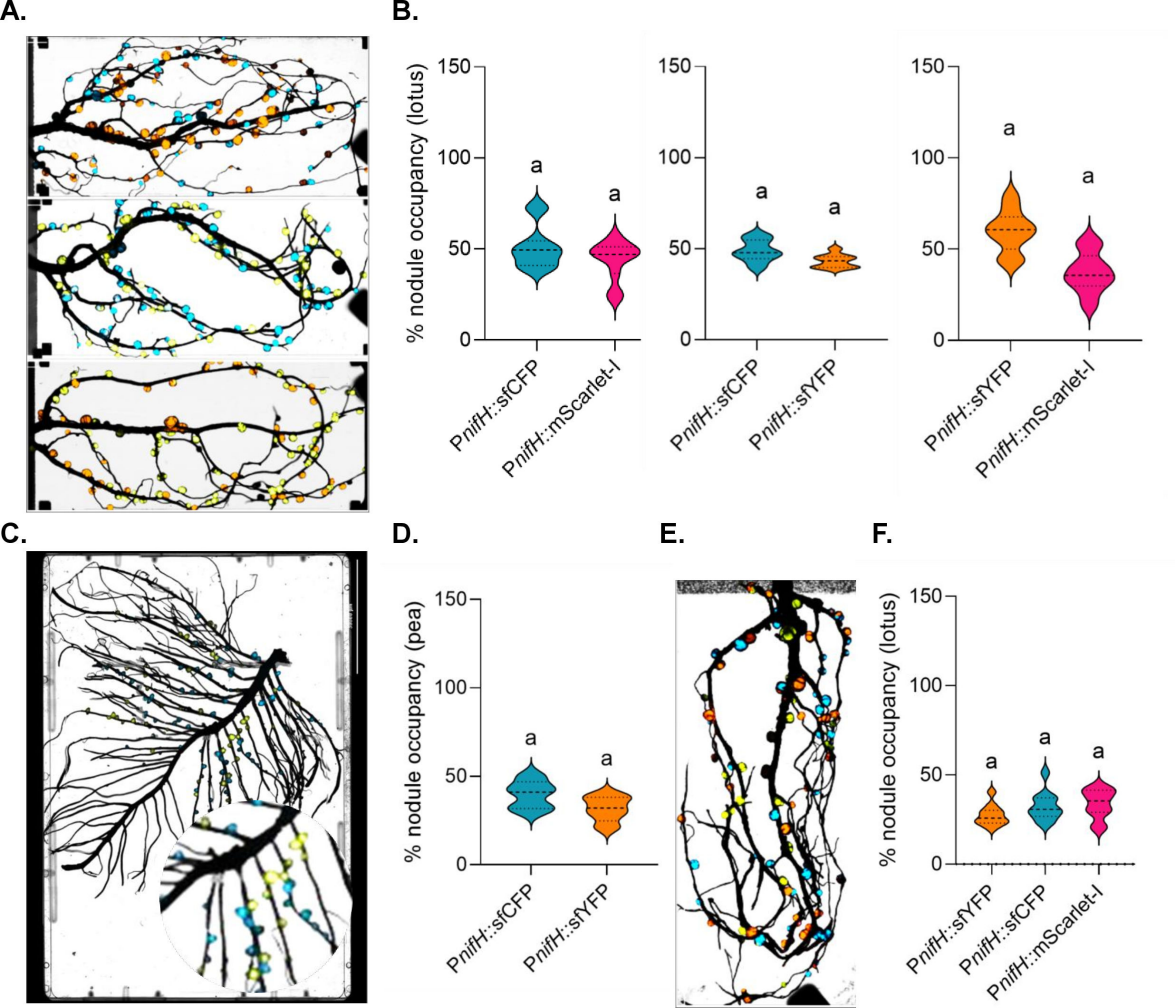
Fluorescent reporters’ expression in co-inoculation experiments. (**A**) *Cytation 5* imaging of *L. japonicus* roots co-inoculated with equal proportions of two uniquely labeled *M. japonicum* strains (sfCFP + mScarlet-I, or sfYFP + sfCFP, or sfYFP + mScarlet-I), each expressing a P*nifH*-driven fluorescent protein. (**B**) Percentage of fluorescently occupied nodules corresponding to the samples shown in panel A. (**C**) *Cytation 5* imaging of *P. sativum* co-inoculated with *R. leguminosarum* strains carrying P*nifH*-driven sfCFP, and sfYFP in a 1:1 ratio. A portion of the image has been zoomed in on a circle for better visualization. (**D**) Percentage of fluorescently occupied nodules corresponding to the samples shown in panel C. (**E**) *Cytation 5* imaging of *L. japonicus* roots co-inoculated with three *M. japonicum* strains (sfCFP + sfYFP + mScarlet-I) in equal proportions, each expressing a P*nifH*-driven fluorescent protein. (**F**) Percentage of fluorescently occupied nodules corresponding to the samples shown in panel E. Data represent six to nine independent biological replicates for panels B and F, and five for panel D. Distinct letters above bars denote statistically significant differences (*P* < 0.05) based on one-way ANOVA followed by Tukey’s HSD test.

A key functionality of symbiosis bioreporters is to facilitate strain identification and visualization of competitiveness for nodule formation during multi-strain co-inoculation. To determine whether P*nifH*-driven fluorescent reporters influence nodule occupancy of uniquely labeled rhizobia, we compared nodule formation by the *M. japonicum* wild-type strain R7A labeled with unique fluorescent proteins, sfCFP, sfYFP, or mScarlet-I inoculated in equal ratios. When pairs of differently labeled strains were introduced together (sfCFP/sfYFP, sfCFP/mScarlet-I, or sfYFP/mScarlet-I), the percentage of nodule occupancy remained similar between the fluorescent labels (Fig. 4A and B). Even in triple inoculations with all three distinct fluorescent tags (sfCFP + sfYFP + mScarlet-I), no single reporter-containing wild-type outcompeted the others, and quantification of fluorescent signal confirmed balanced representation (Fig. 4E and F). Nodules with mixed infections were readily observed at ~10% in all combinations (Fig. S6A), in line with previous estimates (54, 55). Altogether, these findings suggest that reporter identity does not influence competitiveness in this system and that up to three distinct fluorescently labeled strains can be used simultaneously to assess nodule occupancy by *Mesorhizobia* in lotus.

We did similar experiments with pea*-R. leguminosarum* using the wild-type strain Rlv3841 bearing bioreporters. Because P*nifH*::mScarlet-I yielded weaker, inconsistent signals in pea, we restricted analysis in this system to sfCFP and sfYFP. Similar to independent inoculations (Fig. 2A and C), there was not a significant difference in nodule occupancy between the two fluorescent labels (Fig. 4C and D). However, mixed-infection nodules were not observed (Fig. S6B) as readily as in *L. japonicus*.

### Applications of PnifH-driven fluorescent reporters in pea-rhizobium symbiosis

**Fig 5:**
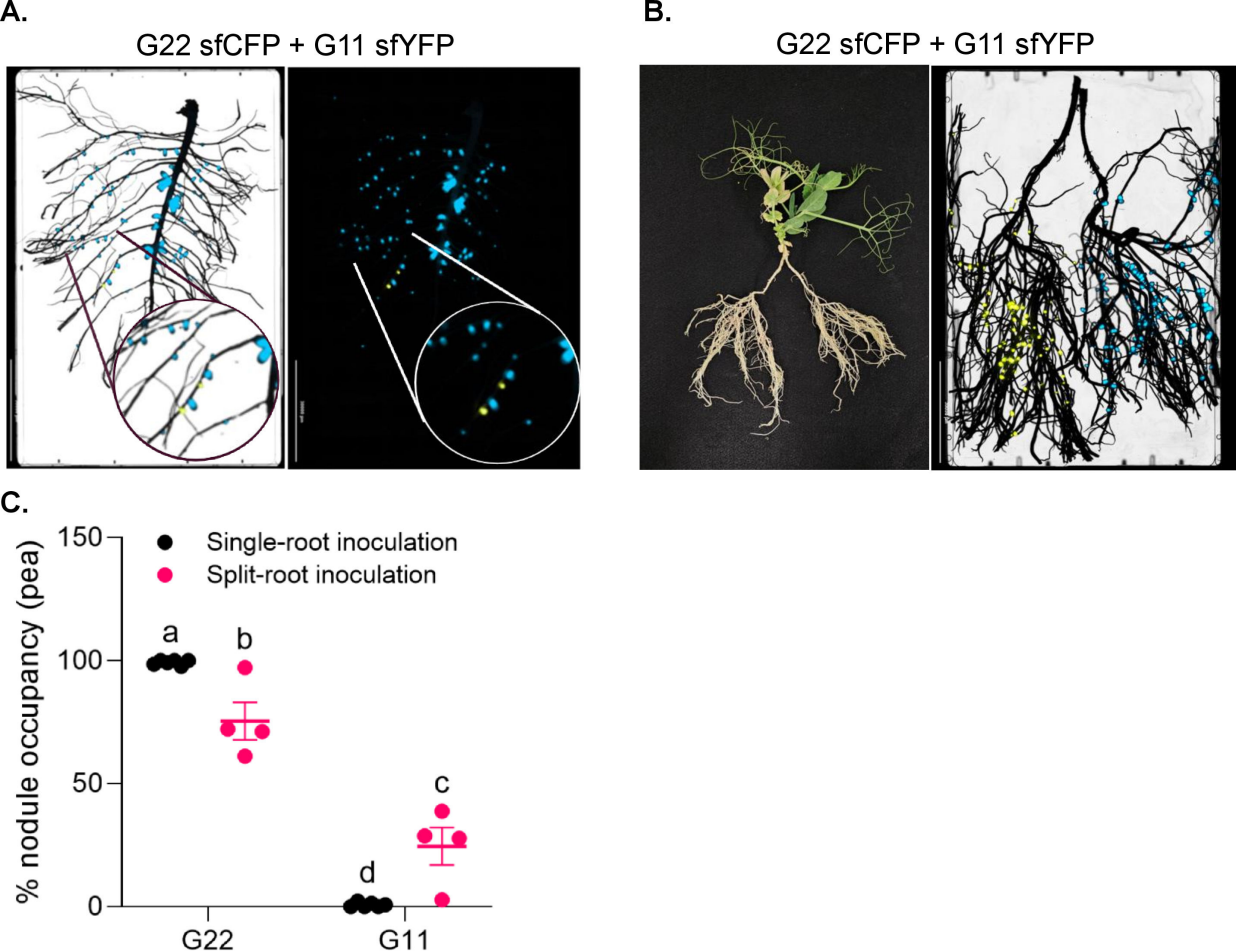
Applications of P*nifH*::fluorescent reporters in *P. sativum-Rhizobium* symbiosis. (**A**) *Cytation 5* imaging of pea roots co-inoculated with equal proportions of two *Rhizobium* strains: G22 (P*nifH*::sfCFP) and G11 (P*nifH*::sfYFP), monitored within a single-root system. A portion of the image has been zoomed in on a circle for better visualization. (**B**) *Cytation 5* imaging of split-root plants inoculated separately with *Rhizobium* strain G22 (P*nifH*::sfCFP) on one root half and strain G11 (P*nifH*::sfYFP) on the other. (**C**) % nodule occupancy across single-root (A) and split-root (B) systems. Data represent five independent biological replicates. Distinct letters above the plots indicate statistically significant differences (*P* < 0.05) as determined by two-way ANOVA followed by Tukey’s HSD test.

A key utility of strain-specific bioreporters is to serve as neutral markers suitable for comparative and ecological studies (54–56). Therefore, we wished to evaluate the efficacy of the reporters described herein in these contexts. To do so, we extended this approach to assess competitiveness among naturally occurring *Rhizobium* by differentially labeling two North Dakota (USA) isolates: G22 with P*nifH*::sfCFP and G11 with P*nifH*::sfYFP. When co-inoculated onto pea roots in single-root set-up in equal proportions, the two strains were readily distinguished based on their fluorescence (Fig. 5A). Consistently, strain G22 displayed a strong competitive advantage, occupying the majority of nodules (Fig. 5A).

To examine strain behavior in the absence of direct competition on the same root system, a split-root assay (Fig. 5B; Fig. S2) was performed. Each half root was inoculated separately with either the sfCFP or sfYFP-labeled strain. Both reporters were easily visualized, and no cross-colonization between root halves was detected (Fig. S2;Fig. 5). Notably, in the split-root setup, the nodule occupancy of G22 exhibited a significant reduction, whereas that of G11 showed a significant increase relative to their respective values observed in the single-root setup (Fig. 5C). Together, these experiments demonstrate that P*nifH*::fluorescent reporters provide a robust tool to monitor strain competitiveness, nodule occupancy, and spatial distribution in both singleroot and split-root experimental systems.

### Deep learning-based quantification of strain effectiveness and competitiveness in split-root assays

**Fig 6:**
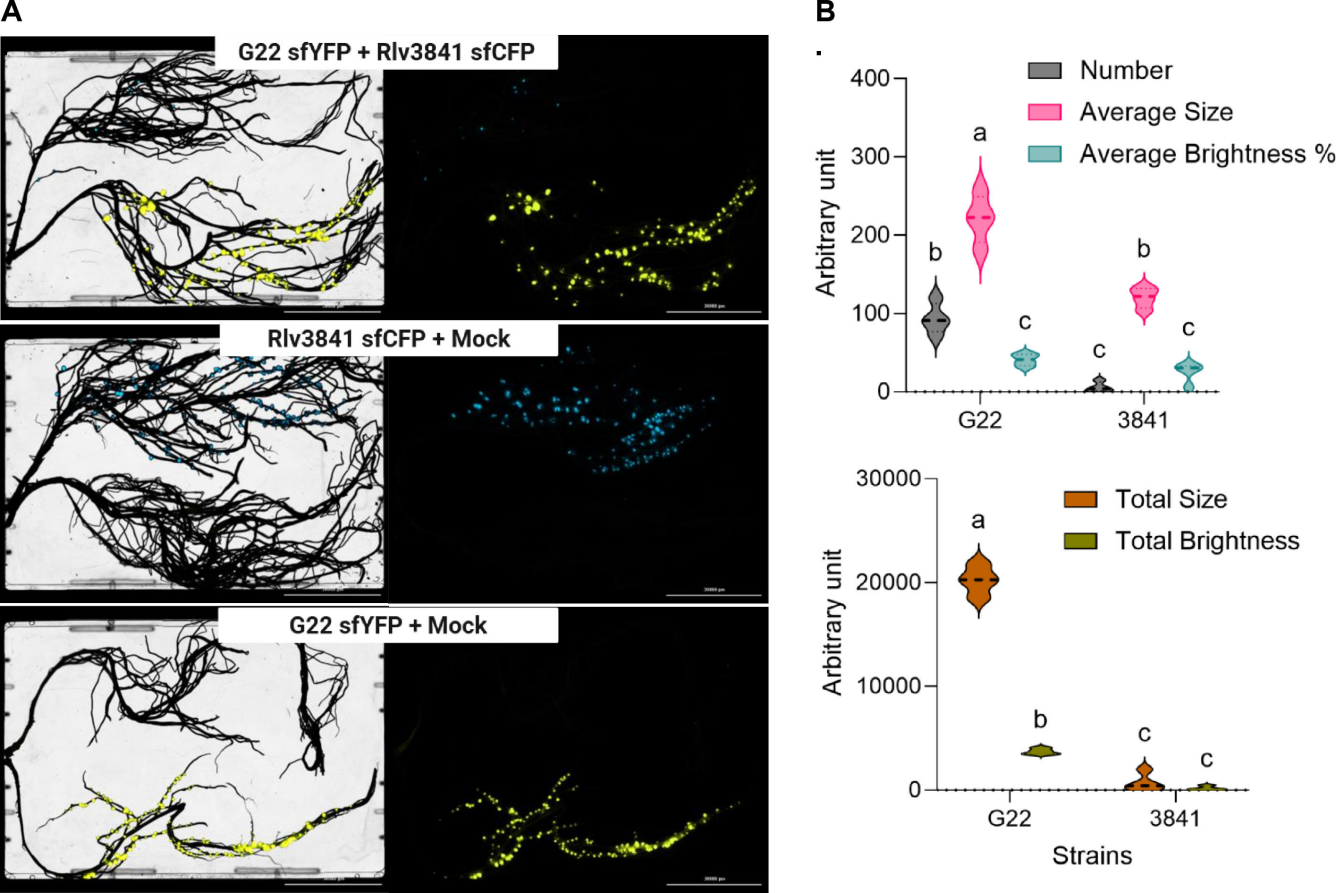
Deep-learning based quantification of nodulation traits in *P. sativum-Rhizobium* symbiosis. (**A**) *Cytation 5* imaging of split-root plants inoculated with *Rhizobium* strains G22 (P*nifH*::sfYFP) and Rlv3841/3841 (P*nifH*::sfCFP). (**B**) Deep learning-based quantification of nodulation traits from split-root experiments shown in panel A. Data represent four independent biological replicates. Distinct letters above bars indicate statistically significant differences (*P* < 0.05) based on two-way ANOVA followed by Tukey’s HSD test.

A central aspect of characterizing legume-rhizobium interactions is the quantification of nodulation traits, including nodule number, size, area, and spatial distribution of nitrogen-fixing nodules. To further benchmark its competitive performance, we compared strain G22, labeled with P*nifH*::sfYFP, against *R. johnsonii* bv. *viciae* 3841, labeled with P*nifH*::sfCFP. Co-inoculation in a pea split-root setup (Fig. S2) enabled clear visualization of both fluorescent markers with no evidence of cross-contamination (Fig. 6A). This system, thus, provided a reliable platform for controlled comparisons of strain competitiveness. Across these assays, G22 again demonstrated a clear competitive advantage, occupying a majority of nodules over Rlv3841 (Fig. 6A). To quantitatively assess competitiveness and nodule parameters, we employed a DL-based image analysis pipeline to extract nodulation parameters, including nodule number, fluorescence intensity, and average size. The outputs of the DL tool revealed consistent trends, as summarized in Fig. 6B, demonstrating a vastly superior nodule formation ability of G22 relative to the reference wild-type strain Rlv3841, with a higher average nodule size and number. Meanwhile, the average fluorescence levels of nodules were similar between those formed by the two strains.

To validate these automated results, we performed parallel quantification using conventional ImageJ-based analysis. The two approaches yielded highly concordant results, underscoring the robustness and accuracy of the DL method for nodulation trait analysis (Fig. S7). Together, these findings establish DL-driven quantification as a reliable and scalable approach for monitoring nodulation outcomes in split-root experiments, while also highlighting the power of P*nifH*::fluorescent reporters for tracing strain performance in pea*-Rhizobium* symbioses.

## DISCUSSION

This study established newly optimized bioreporter systems for investigating legume-rhizobium symbiosis by integrating promoter-driven fluorescent reporters with automated quantification of nodulation traits. Our systematic evaluation of various P*nifH*- and P*uni*-controlled expression of fluorescent proteins across two phylogenetically distinct symbioses provided significant insights into the utility of these reporter systems for studying symbiotic nitrogen fixation. The utilization of the *nifH* promoter to control reporter expression ensures effects of free-living reporter expression do not convolute measurements of competition for nodule occupancy. Moreover, it also allows the fluorescent protein signal to act as a bioreporter of nitrogenase activity (22), the utility of which is enhanced by the concurrent measurement of bioreporter activity in multiple strains on the same root. Additionally, the introduction of a high-throughput segmentation pipeline enables robust, quantitative, and non-destructive visualization of nodulation parameters, opening new possibilities for efficient and comprehensive analysis.

The P*nifH* promoter-based reporter system has become a standard tool in the study of legume-rhizobium symbiosis, reliably serving as a proxy for nitrogenase activity and as an indicator of rhizobial nodule colonization and formation of functional bacteroids (22, 57, 58). Our results indicate that P*nifH*-driven expression of sfYFP and sfCFP yields robust and reproducible fluorescence in both determinate (lotus) and indeterminate (pea) nodules, facilitating precise visualization of strain distribution, nodule occupancy, and regions of active nitrogenase activity (Fig. 2A through D; Fig. 4A through F; Fig. 5A and B). Since the P*nifH*s used in this study are derived from consensus sequences from a set of rhizobial strains, these promoter-reporters can be used in a wide range of *Mesorhizobium* and *Rhizobium* strains. Our finding reinforces the utility of P*nifH*-based fluorescent bioreporters as valuable tools for evaluating the effectiveness and competitiveness of rhizobial symbionts within diverse legume hosts.

However, not all fluorophores performed uniformly across host systems. mScarlet-I exhibited strong and consistent performance in lotus nodules (Fig. 2B and D). In contrast, its expression in pea nodules was inconsistent, with approximately 20% of nodules lacking an easily detectable signal (Fig. 2A and C). This was despite mScarlet-I being a monomeric protein (39, 59), which is known to be highly stable in other biological systems. Efforts to improve mScarlet-I readability in pea nodules by adjusting imaging conditions often increased background autofluorescence, further limiting the interpretability of the results. Similar inconsistencies were observed with other RFPs, both monomeric (mRFP1, mARs1) and multimeric (Azami Red), none of which provided reliable detection in pea (Fig. 3A). These findings highlight a critical limitation in using RFPs, indicating that host-specific factors, such as the intracellular environment and nodule physiology, significantly influence reporter performance and stability. Although monomeric fluorescent proteins are generally preferred for their reliable *in vivo* expression in biological systems (39, 59), the present results demonstrate that their effectiveness can be species-dependent, particularly for RFPs in pea.

To further examine reporter versatility, we tested the P*uni* promoter-based reporter system in both the legume systems, a constitutive σ−70 consensus promoter element designed for broad expression regardless of nodulation status (53, 60, 61). While P*uni*-driven fluorophores enabled analysis independent of microenvironmental cues specific to nodules, they brought limited benefits. In pea, P*uni* drove sfYFP, sfCFP, and mScarlet-I at levels similar to P*nifH*, but did not improve the reliability of RFP detection, as signals were weak and inconsistent (Fig. 3A and B; Fig. S4). Although more nodules were detected with Azami Red and monomeric mRFP1, their fluorescence was still low and variable (Fig. 3A and B). Expression from the P*uni* promoter was very weak for all fluorophores in lotus, pointing toward a host-dependent variability in the expression of constitutive promoters (Fig. S5C). In indeterminant nodules of pea, where the P*uni* promoter was functional, a gradual transition between the expression of the housekeeping σ−70 *rpoD* and the σ factor associated with expressing nitrogen-fixation genes, σ−54 *rpoN,* can be observed between zones of the nodule (62). The weak expression from the P*uni* σ−70 promoter in lotus nodules may reflect a more uniform downregulation of σ−70-based gene expression within inhabitants of determinant nodules. This may have implications for promoter design for use within rhizobium bacteroids. However, it is also essential to consider that the strength of the Anderson library-derived constitutive P*uni* promoter can be further enhanced through selection of stronger variants, which may impact the efficacy of labeling in lotus nodules (43, 63, 64). Overall, these patterns underscore that the biochemical properties of both the chosen fluorescent protein and the host cellular milieu are as critical as the promoter in achieving reliable and interpretable reporter output.

Despite forming functional nodules, host-specific factors such as oxygen availability, protein folding, and chromophore maturation variables may affect fluorescent protein readouts. For instance, it is known that the maturation of GFP and RFP derivatives is oxygen-dependent (35, 65, 66). Consequently, the variability of oxygen within determinate and indeterminate nodule types may contribute to weak or inconsistent fluorescence. Determinate nodules, like those in lotus, possess an oxygen diffusion barrier at the apex, which maintains uniformly low oxygen levels throughout the nodule (67). By contrast, indeterminate nodules lack this apical barrier, resulting in a longitudinal gradient of oxygen concentration (68, 69). We speculate that the host-specific challenges we observed with RFPs may result from differences in the oxygen levels in the pea and lotus nodules we tested in this study. This limitation, due to the oxygen-dependent maturation of the fluorophores used in our study, could potentially be addressed by employing flavin-based fluorescent proteins (FbFPs), which mature independently of oxygen (70). However, FbFPs currently suffer from reduced brightness, a limited color spectrum, and relatively few biological validations of performance, which may constrain their utility in nodule imaging (71, 72). Another emerging class of oxygen-independent fluorophores is the small Ultra-Red Fluorescent Protein (smURFP) family (73, 74). Although their performance in biological systems remains relatively unexplored (75–77), smURFPs represent a promising option for imaging rhizobia within root nodules.

We observed other host-specific differences, including a rarer occurrence of mixed nodules in pea relative to lotus, as well as a greater proportion of “dark” nodules (Fig. S6A and B). We expected to observe a rare but significant population of mixed nodules in both systems, based on previous studies in pea (22, 54), and lotus (78). It is possible that the P*nifH* fluorescence signal is ineffective at quantifying mixed populations in indeterminant nodules due to a biased expression in the nitrogen-fixing zone of the nodule, with the mixed strains variably occupying distinct nodule zones (54). In pea, approximately 25% of nodules also did not display any fluorescent signals despite successful nodulation (Fig. S6B). The underlying cause of this observation remains unclear, but it may be associated with rhizobia exhibiting weak nitrogenase activity, potentially influenced by the anatomical and developmental characteristics of indeterminate nodules (79). In these nodules, the nitrogen-fixing zone is located at a distance from the meristem, resulting in a delay in the maturation of newly formed cells and a lag before rhizobia can initiate nitrogen fixation (41, 80). In contrast, smaller determinate nodules develop more synchronously, leading to a more uniform and consistent rate of nitrogen fixation (41, 80). This difference in developmental progression also results in uniform senescence of determinate nodules, whereas indeterminate nodules exhibit gradual senescence (81). Overall, these host-dependent effects reinforce the need to empirically validate fluorescent systems in each legume species, rather than assuming cross-compatibility.

Overall, our data indicate that P*nifH-*driven fluorescent proteins offer significant opportunity for studying rhizobium-legume symbiosis, with particularly promising performance within lotus nodules formed by *Mesorhizobium*. Despite the host-specific challenges we discuss above within the pea-*Rhizobium* system, we still demonstrated substantial utility of P*nifH-*sfYFP and P*nifH*:sfCFP reporters. We exemplified this utility by monitoring the fluorescence of nodules and nodulation characteristics of two natural *Rhizobium* strains in both single-inoculation (Fig. 5A) and split-root configurations in pea (Fig. 5B), where we observed different patterns of competitive interaction (Fig. 5C). The quantification of these differences with the same set of rhizobia facilitates investigations that separate local microbe-microbe competitive interactions from plant partner choice influence on rhizobial competitiveness (82). In single-root inoculation experiments, strain G22 was highly competitive, occupying the majority of nodules (Fig. 5A and C). In contrast, under split-root conditions, where G22 and G11 were not in direct contact but exposed to shared systemic signals, nodule occupancy for G11 was significantly higher compared to its single-root inoculation condition (Fig. 5B and C). These findings underscore the significance of experimental context in assessing competitiveness and suggest that split-root assays should be incorporated as a complementary approach for a robust evaluation of rhizobial competition under diverse conditions.

Recently, the adoption of machine learning and deep learning approaches in plant biology research has accelerated markedly, particularly for quantitative trait analysis and automated phenotyping. Nonetheless, reports focusing specifically on the quantification of nodulation traits in legumes using these computational tools remain scarce. The majority of research in this domain focuses on high-throughput phenotyping platforms that characterize nodulation using image-based methods, typically applied to nodules naturally infected by rhizobia, and does not employ fluorescent bioreporters or tagged strains (83–87). To the best of current knowledge, this study represents the first report on the application of DL for analyzing fluorescent imaging data directly linked to the nitrogen fixation status of legume nodules in real time. Automated, DL-based quantification of nodule traits was integrated into split-root competition assays, enabling high-throughput and reproducible analysis that closely paralleled conventional ImageJ-based measurements, thereby validating the utility of these automated pipelines for phenotyping nodulation and symbiotic performance (Fig. 6A and B; Fig. S7).

Although P*nifH*-driven sfYFP and sfCFP are validated as robust tools for real-time symbiosis monitoring and for studying the effectiveness and competitiveness of rhizobium strains within the host, several caveats remain. Reporter performance is sensitive to physiological parameters such as oxygen, pH, and protein folding environment, with these constraints most notably affecting RFP performance in pea. Rare co-infection events indicate potential complexity in interpreting nodule occupancy, particularly in assays involving multiple strains. While automated imaging significantly improves throughput and reproducibility, ongoing benchmarking against standard approaches is necessary to ensure data robustness and accuracy. This effort is further constrained by the limited reference literature on the use of deep learning for quantifying nodulation traits.

While the reporter systems here show apparent efficacy for investigating rhizobium-legume symbiosis with fluorescent bioreporters, future optimization of fluorescent proteins, especially for red and far-red channels, will be essential to fully realize the potential of multiplexed visualization in diverse legumes. Integrating these improved reporters with conventional assessments and next-generation sequencing may provide a deeper understanding of plant-microbe interactions and symbiotic regulation. The combination of reliable promoter-reporter systems with scalable, automated quantification establishes a strong foundation for high-resolution, mechanistic studies of symbiotic nitrogen fixation and host-microbe ecology.

## SUPPLEMENTAL MATERIAL

Supplemental material aem.02154-25-s0001.docx Figures S1 to S7; Tables S1 to S7.

ASM does not own the copyrights to Supplemental Material that may be linked to, or accessed through, an article. The authors have granted ASM a non-exclusive, world-wide license to publish the Supplemental Material files. Please contact the corresponding author directly for reuse.
