# Supplementary material for: Host-specific fluorescence dynamics in legume-rhizobium symbiosis during nodulation
Source: Appl Environ Microbiol. 2026 Jan 16;92(2):e02154-25. doi: 10.1128/aem.02154-25 (PMC12915343; doi:10.1128/aem.02154-25)
Supplement: Supplemental material — Figures S1 to S7; Tables S1 to S7. [file aem.02154-25-s0001.docx]

**Supplementary Figures:**


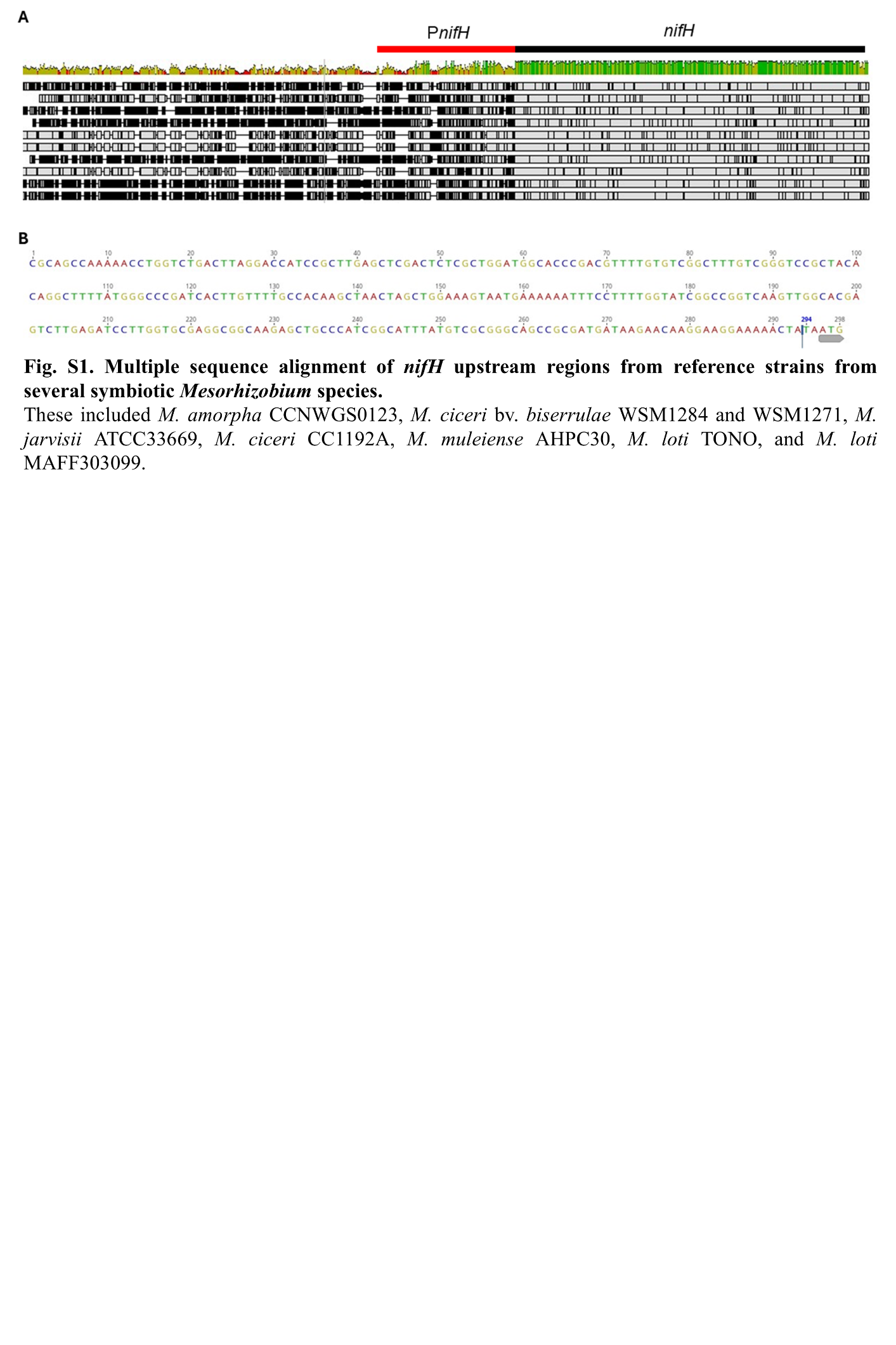


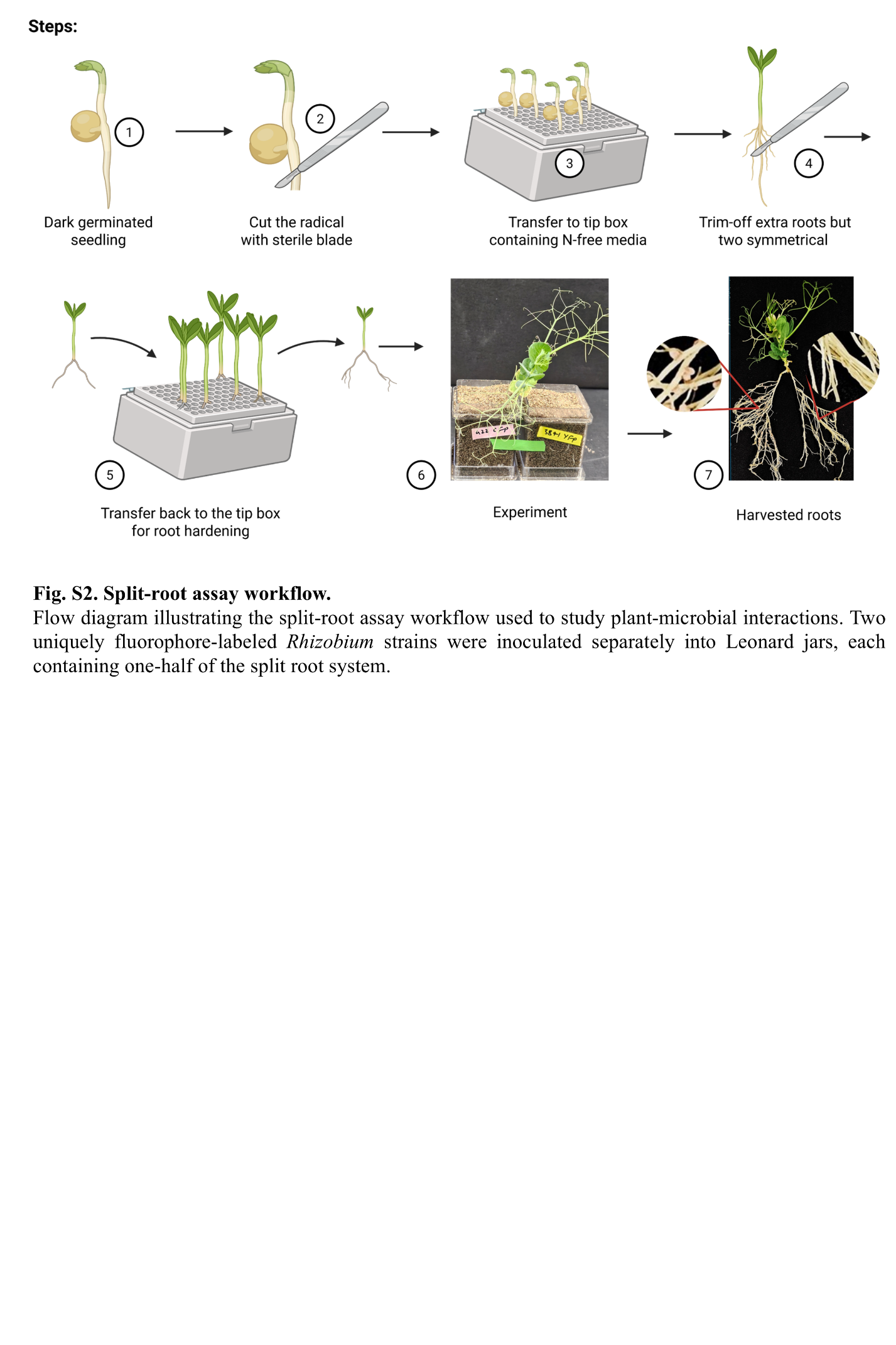

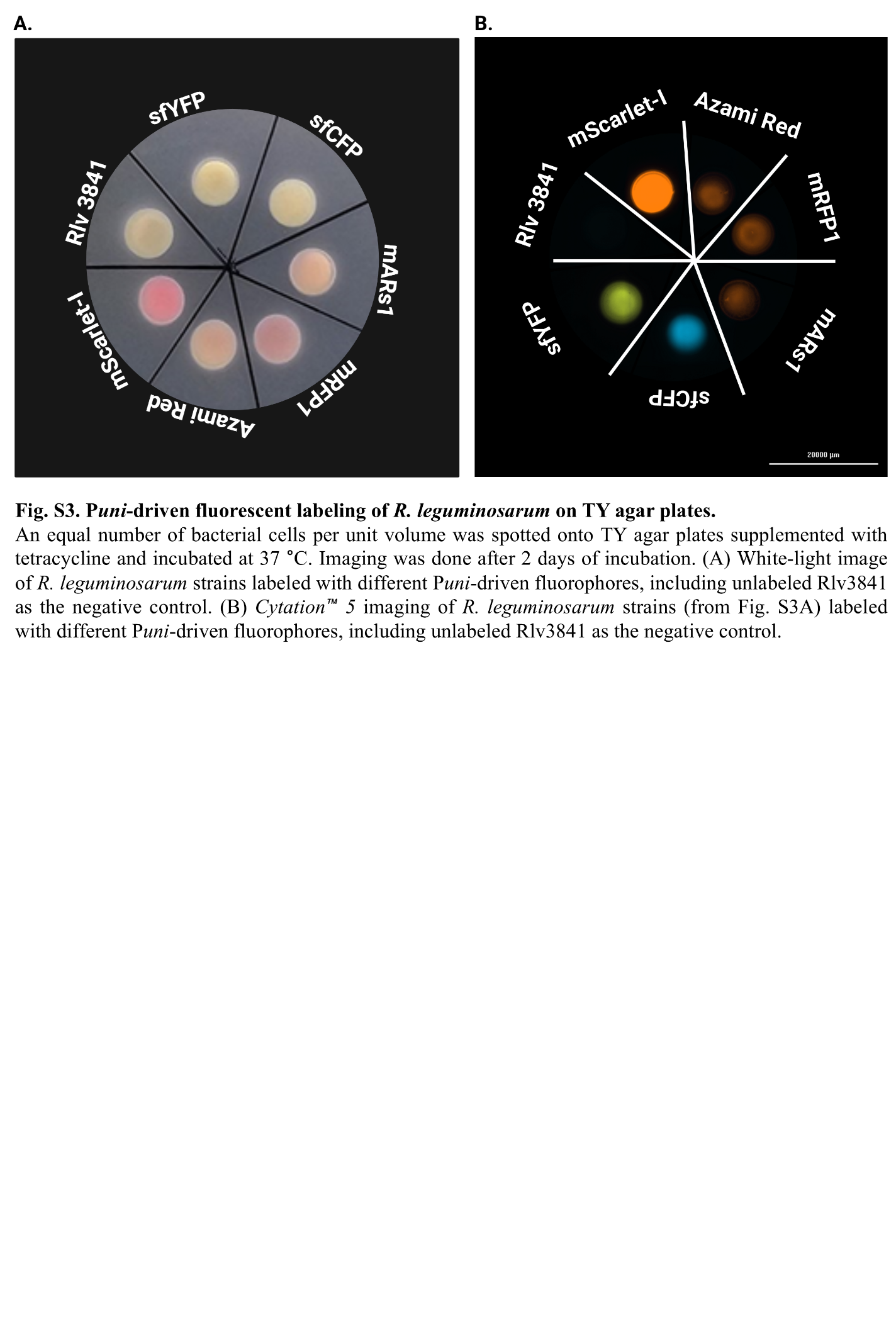

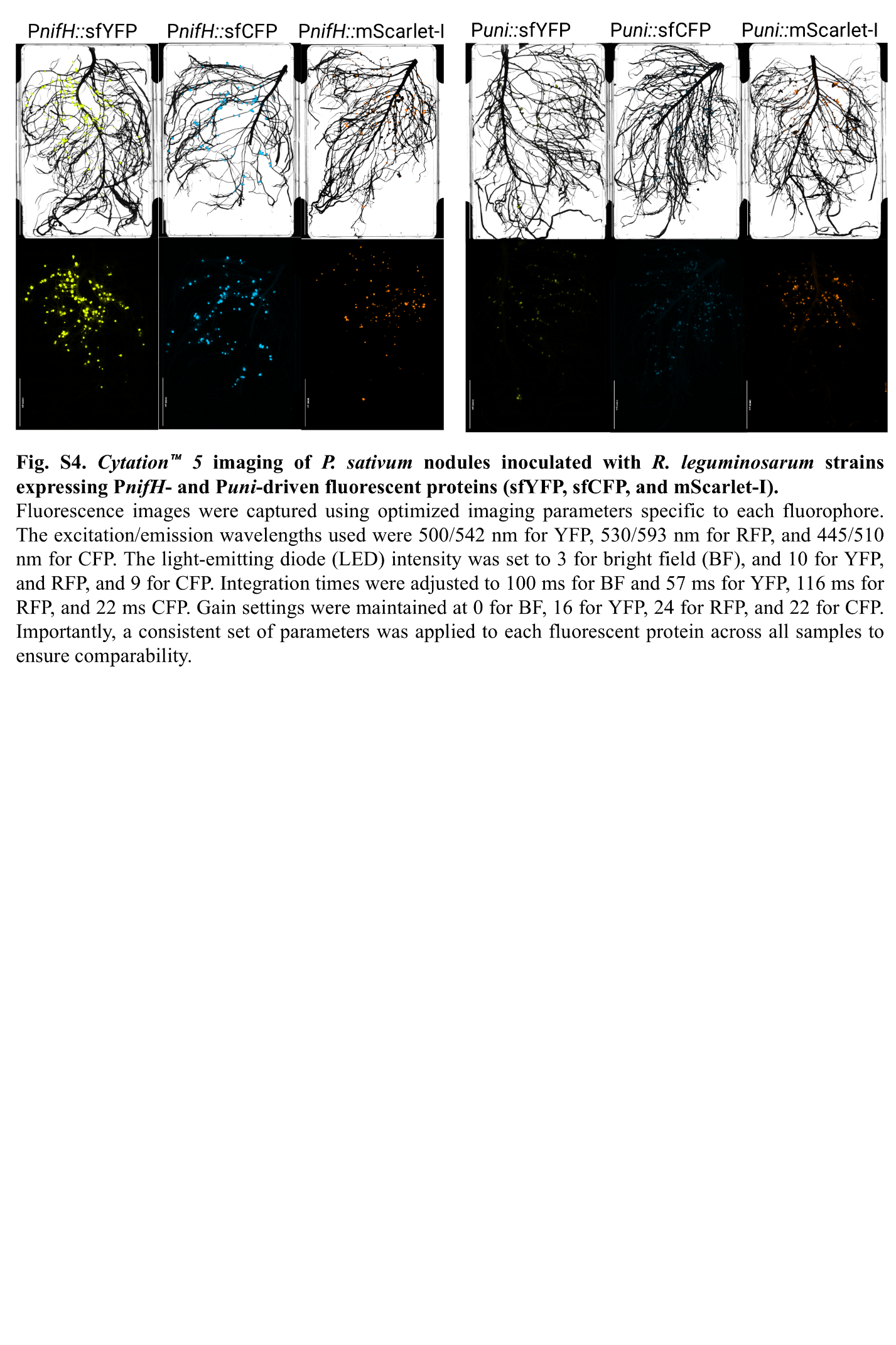


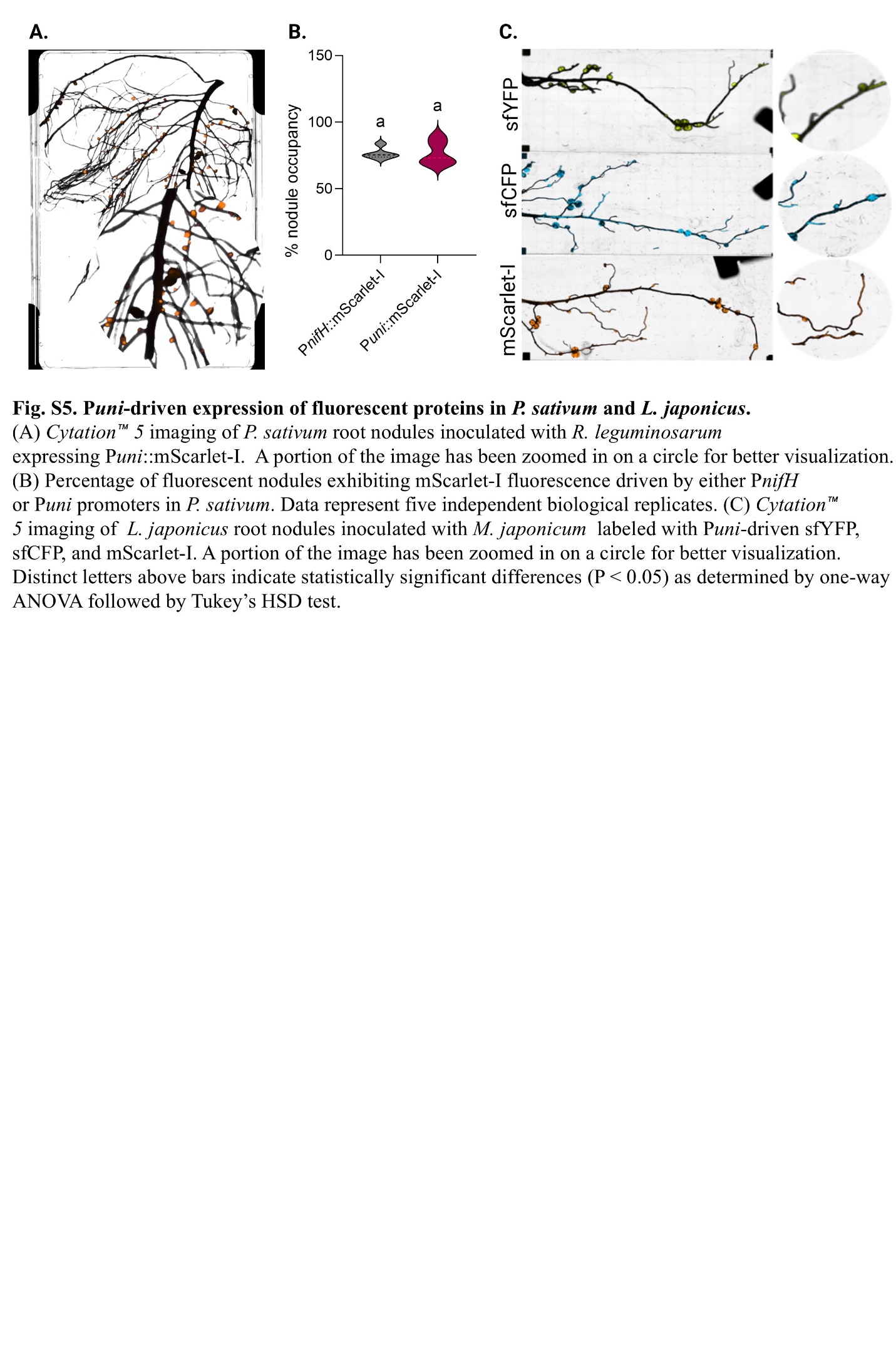

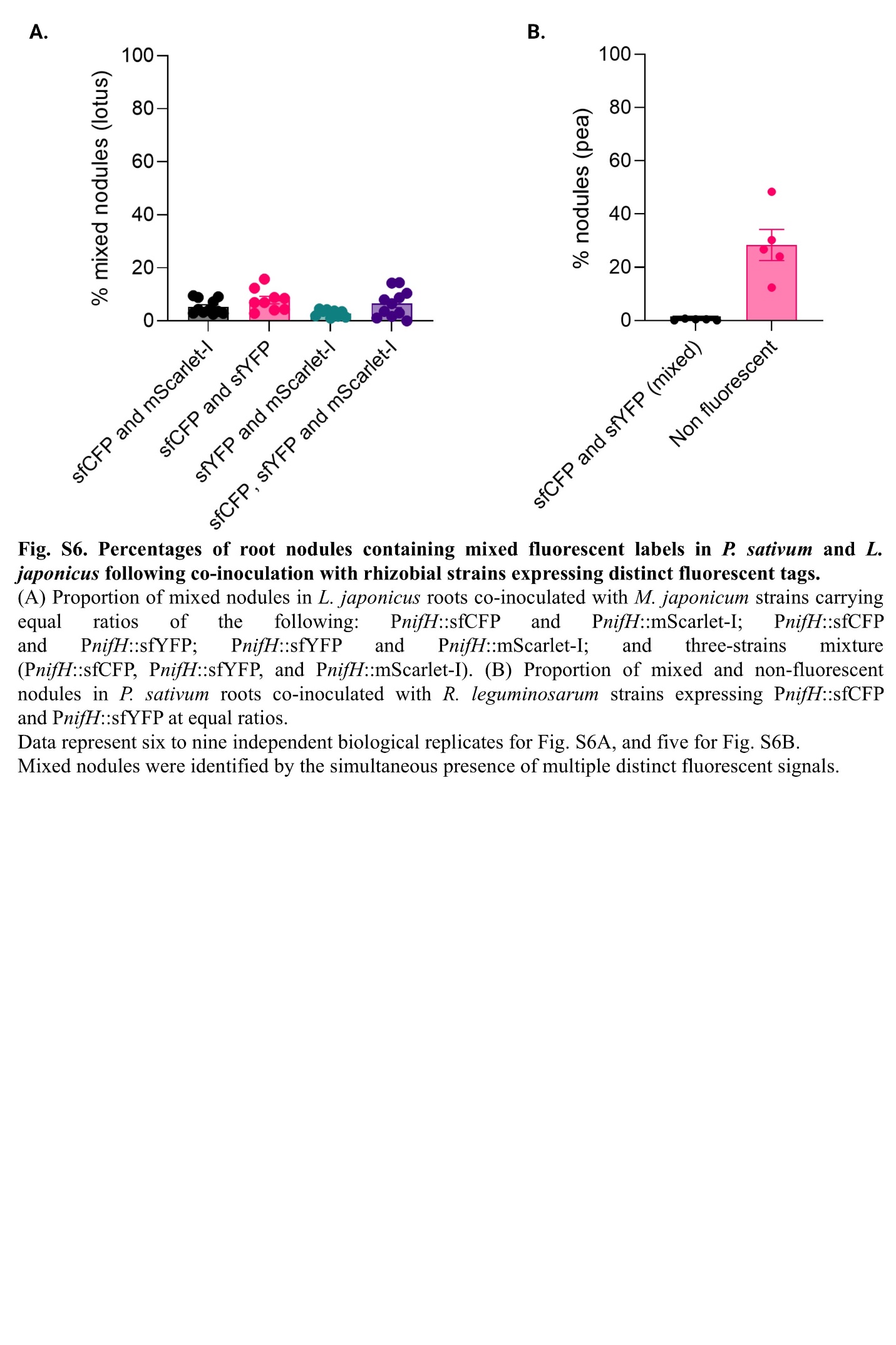


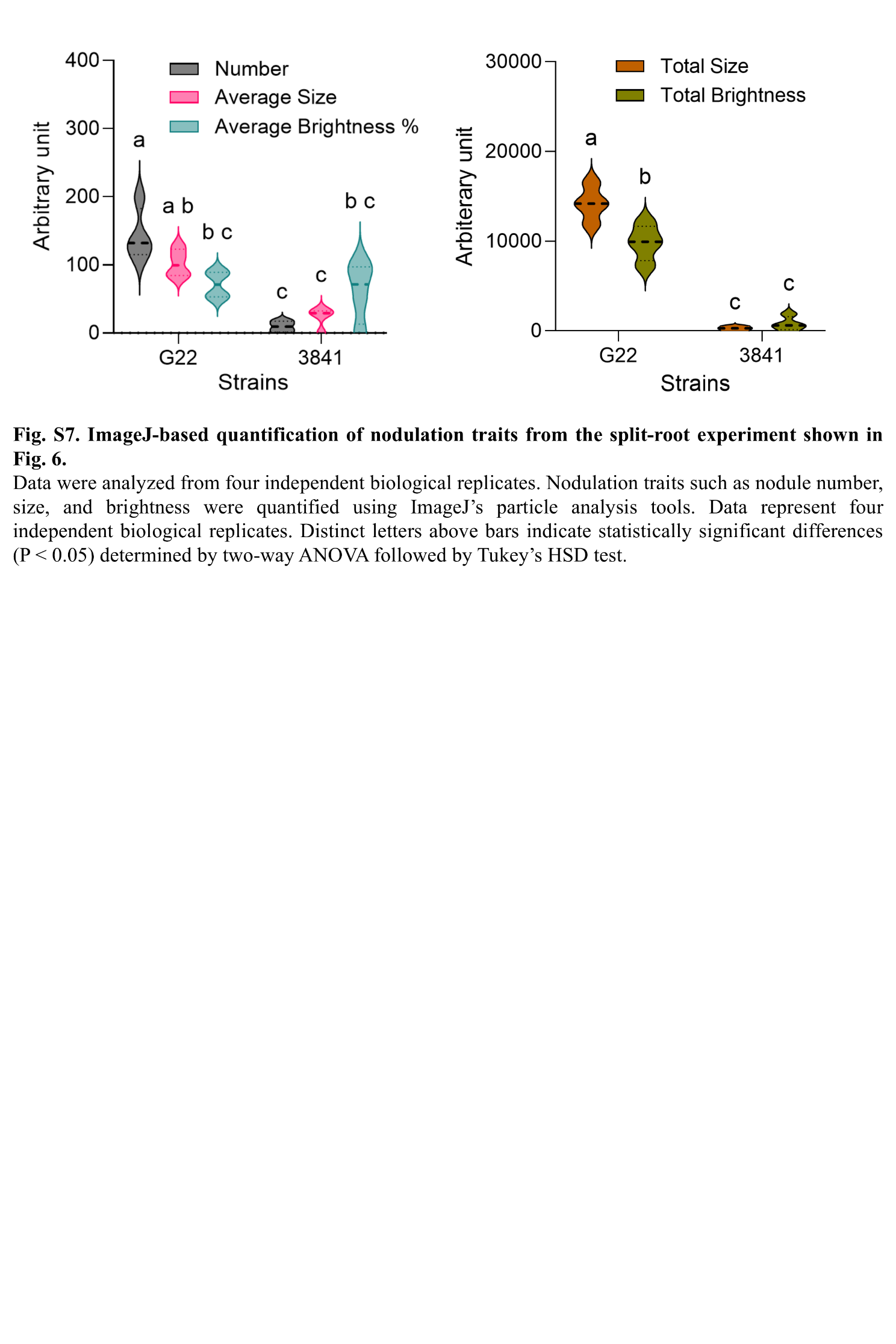


**Supplementary Tables:**

**Table S1:** Details of the plasmid components and constructs, and plasmid-engineered rhizobia used in this paper. Further details can be found in the Microbiology Resource Announcement (MRA) manuscripts by Senanayake et al., 2025, and Pease et al., 2025.

| **Name** | **Description** | **Source** | **Addgene Plasmid #** |
| --- | --- | --- | --- |
| C3m | superfolder green fluorescent protein (sfGFP), Golden Gate component; Addgene CIDAR MoClo Vol.1 Extension (kit #1000000161); Amp^R^ | Richard Murray Lab: CIDAR MoClo Extension Unpublished | 120956 |
| C5m | monomeric red fluorescent protein; mRFP1, Golden Gate component; Addgene CIDAR MoClo Vol.1 Extension (kit #1000000161); Amp^R^ | Richard Murray Lab: CIDAR MoClo Extension Unpublished | 120957 |
| C51m | superfolder yellow fluorescent protein (sfYFP), Golden Gate component; Addgene CIDAR MoClo Vol.1 Extension (kit #1000000161); Amp^R^ | Richard Murray Lab: CIDAR MoClo Extension Unpublished | 120975 |
| C91m | superfolder cyan fluorescent protein (sfCFP), Golden Gate component; Addgene CIDAR MoClo Vol.1 Extension (kit #1000000161); Amp^R^ | Richard Murray Lab: CIDAR MoClo Extension Unpublished | 120999 |
| C99m | mScarlet-I, Golden Gate component; Addgene CIDAR MoClo Vol.1 Extension (kit #1000000161); Amp^R^ | Richard Murray Lab: CIDAR MoClo Extension Unpublished | 121003 |
| J23106 | J23106 Anderson library promoter, Golden Gate component; Addgene CIDAR MoClo Parts Kit (kit #1000000059); Amp^R^ | (41) | 65992 |
| BCD12 | BCD12 bicistronic ribosome binding cite, Golden Gate component; Addgene CIDAR MoClo Parts Kit (kit #1000000059); Amp^R^ | (41) | 66023 |
| pOGG043 | Synthetic *Rhizobium* P*nifH* Golden Gate component Rlv_Ps*nifH*; Sp^R^ | (22) | 133123 |
| pNDGG003 | BEVA 2.0 level 1 Golden Gate cloning vector with Bsa1 exchangeable lacZ, RK2 origin of replication, and par stability, Tc^R^ | (50) | 231316 |
| pNDGG004 | BEVA 2.0 level 1 Golden Gate cloning vector with Bsa1 exchangeable lacZ, pBBR1origin of replication, and par stability, Tc^R^ | (50) | 231317 |
| pNDGG037 | BEVA 2.0 T2m terminator with DF extension for CIDAR MoClo GG; Sp^R^ | (50) | 231337 |
| pNDGG076 | Synthetic *Mesorhizobium* P*nifH* PU/AB Golden Gate component Meso_Ps*nifH*; Amp^R^ | This work |  |
| pNDGG078 | mARs1 SC/CD Golden Gate component; Amp^R^ | This work |  |
| pNDGG079 | AzamiRed1.0 SC/CD Golden Gate component; Amp^R^ | This work |  |
| pNDMS054 | Level 1 Golden Gate Cloning with pNDGG003 as backbone with parts: J23106-BCD12-sfGFP-T2m; Tc^R^ | (50) |  |
| pNDMS055 | Level 1 Golden Gate Cloning with pNDGG003 as backbone with parts: J23106-BCD12-sfCFP-T2m; Tc^R^ | (50) |  |
| pNDMS056 | Level 1 Golden Gate Cloning with pNDGG003 as backbone with parts: J23106-BCD12-sfYFP-T2m; Tc^R^ | (50) |  |
| pNDMS057 | Level 1 Golden Gate Cloning with pNDGG003 as backbone with parts: J23106-BCD12-mScarlet-I-T2m; Tc^R^ | (50) |  |
| pNDMS156 | Level 1 Golden Gate Cloning with pNDGG003 as backbone with parts: Meso_Ps*nifH*-sfGFP-T2m; Tc^R^ | Accompanying manuscript Senanayake et al. 2025 |  |
| pNDMS157 | Level 1 Golden Gate Cloning with pNDGG004 as backbone with parts: Meso_Ps*nifH*-sfGFP-T2m; Tc^R^ | Accompanying manuscript Senanayake et al. 2025 |  |
| pNDMS237 | Level 1 Golden Gate Cloning with pNDGG004 as backbone with parts: Meso_Ps*nifH*-sfYFP-T2m; Tc^R^ | Accompanying manuscript Senanayake et al. 2025 |  |
| pNDMS238 | Level 1 Golden Gate Cloning with pNDGG004 as backbone with parts: Meso_Ps*nifH*-sfCFP-T2m; Tc^R^ | Accompanying manuscript Senanayake et al. 2025 |  |
| pNDMS239 | Level 1 Golden Gate Cloning with pNDGG004 as backbone with parts: Meso_Ps*nifH*-mScarlet-I-T2m; Tc^R^ | Accompanying manuscript Senanayake et al. 2025 |  |
| pNDMS290 | Level 1 Golden Gate Cloning with pNDGG004 as backbone with parts: Rlv_Ps*nifH*-sfYFP-T2m; Tc^R^ | Accompanying manuscript Pease et al. 2025 |  |
| pNDMS291 | Level 1 Golden Gate Cloning with pNDGG004 as backbone with parts: Rlv_Ps*nifH*-sfCFP-T2m; Tc^R^ | Accompanying manuscript Pease et al. 2025 |  |
| pNDMS292 | Level 1 Golden Gate Cloning with pNDGG004 as backbone with parts: Rlv_Ps*nifH*-mScarlet-I-T2m; Tc^R^ | Accompanying manuscript Pease et al. 2025 |  |
| pNDMS377 | Level 1 Golden Gate Cloning with pNDGG004 as backbone with parts: J23106-BCD12-sfYFP-T2m; Tc^R^ | This work |  |
| pNDMS378 | Level 1 Golden Gate Cloning with pNDGG004 as backbone with parts: J23106-BCD12-sfCFP-T2m; Tc^R^ | This work |  |
| pNDMS379 | Level 1 Golden Gate Cloning with pNDGG004 as backbone with parts: J23106-BCD12-mScarlet-I-T2m; Tc^R^ | This work |  |
| pNDMS344 | Level 1 Golden Gate Cloning with pNDGG004 as backbone with parts: Rlv_Ps*nifH*-mRFP1-T2m; Tc^R^ | This work |  |
| pNDMS803 | Level 1 Golden Gate Cloning with pNDGG004 as backbone with parts: Rlv_Ps*nifH*-mARs1-T2m; Tc^R^ | This work |  |
| pNDMS805 | Level 1 Golden Gate Cloning with pNDGG004 as backbone with parts: Rlv_Ps*nifH*-AzamiRed1.0-T2m; Tc^R^ | This work |  |
| pNDMS807 | Level 1 Golden Gate Cloning with pNDGG004 as backbone with parts: J23106-BCD12-mRFP1-T2m; Tc^R^ | This work |  |
| pNDMS808 | Level 1 Golden Gate Cloning with pNDGG004 as backbone with parts: J23106-BCD12-mARsI-T2m; Tc^R^ | This work |  |
| pNDMS809 | Level 1 Golden Gate Cloning with pNDGG004 as backbone with parts: J23106-BCD12-AzamiRed1.0-T2m; Tc^R^ | This work |  |
| RmND298 | pNDMS156 conjugated into *M. japonicum* strain R7A; Tc^R^ | This work |  |
| RmND299 | pNDMS157 conjugated into *M. japonicum* strain R7A; Tc^R^ | This work |  |
| RmND533 | pNDMS237 conjugated into *M. japonicum* strain R7A; Tc^R^ | This work |  |
| RmND534 | pNDMS238 conjugated into *M. japonicum* strain R7A; Tc^R^ | This work |  |
| RmND535 | pNDMS239 conjugated into *M. japonicum* strain R7A; Tc^R^ | This work |  |
| RmND696 | pNDMS808 conjugated into *R. leguminosarum* strain Rlv3841; Sm^R^,Tc^R^ | This work |  |
| RmND697 | pNDMS809 conjugated into *R. leguminosarum* strain Rlv3841; Sm^R^,Tc^R^ | This work |  |
| RmND698 | pNDMS807 conjugated into *R. leguminosarum* strain Rlv3841; Sm^R^,Tc^R^ | This work |  |
| RmND700 | pNDMS803 conjugated into *R. leguminosarum* strain Rlv3841; Sm^R^,Tc^R^ | This work |  |
| RmND701 | pNDMS344 conjugated into *R. leguminosarum* strain Rlv3841; Sm^R^,Tc^R^ | This work |  |
| RmND702 | pNDMS805 conjugated into *R. leguminosarum* strain Rlv3841; Sm^R^,Tc^R^ | This work |  |
| RmND718 | pNDMS379 conjugated into *R. leguminosarum* strain Rlv3841; Sm^R^,Tc^R^ | This work |  |
| RmND719 | pNDMS377 conjugated into *R. leguminosarum* strain Rlv3841; Sm^R^,Tc^R^ | This work |  |
| RmND720 | pNDMS378 conjugated into *R. leguminosarum* strain Rlv3841; Sm^R^,Tc^R^ | This work |  |
| RmND623 | pNDMS290 conjugated into *R. leguminosarum* strain Rlv3841; Sm^R^,Tc^R^ | This work |  |
| RmND624 | pNDMS291 conjugated into *R. leguminosarum* strain Rlv3841; Sm^R^,Tc^R^ | This work |  |
| RmND625 | pNDMS292 conjugated into *R. leguminosarum* strain Rlv3841; Sm^R^,Tc^R^ | This work |  |
| RmND635 | pNDMS290 conjugated into *Rhizobium* strain G22; Tc^R^ | This work |  |
| RmND636 | pNDMS291 conjugated into *Rhizobium* strain G22; Tc^R^ | This work |  |
| RmND629 | pNDMS290 conjugated into *Rhizobium* strain G11; Tc^R^ | This work |  |

**Table S2:** Nucleotide sequence of promoters and fluorescent protein encoding genes.

| **Names** | **Sequences** | **References** |
| --- | --- | --- |
| Meso_Ps*nifH* | GGAGCGCAGCCAAAAACCTGGTCTGACTTAGGACCATCCGCTTGAGCTCGACTCTCGCTGGATGGCACCCGACGTTTTGTGTCGGCTTTGTCGGGTCCGCTACACAGGCTTTTATGGGCCCGATCACTTGTTTTGCCACAAGCTAACTAGCTGGAAAGTAATGAAAAAATTTCCTTTTGGTATCGGCCGGTCAAGTTGGCACGAGTCTTGAGATCCTTGGTGCGAGGCGGCAAGAGCTGCCCATCGGCATTTATGTCGCGGGCAGCCGCGATGATAAGAACAAGGAAGGAAAAACTATAATG | This paper, Senanayake et al., 2025 |
| Rlv_Ps*nifH* | GGAGCTCGACACGACATTGTCGTCACCTTTGTCGGGTCCGCGACAAGCTTTTTGTTCCTCGATCCCGTATGTTTTCGCCTAAATATCTGAAACAGCAGCAAATTATTTTTCGTTAGCTCAATCGGCCCACTTGGCACGAGTCTTGAAATTTATTGCGAGGCGGCGGAGCTGCCGGCCGCATCCGTGCGGCATAACCACGGTTGTGAACAAATGAAGGAAGGCCAAATG | (Mendoza-Suárez et al., 2020) |
| P*uni* | GGAGTTTACGGCTAGCTCAGTCCTAGGTATAGTGCTAGCTACTGGGCCCAAGTTCACTTAAAAAGGAGATCAACAATGAAAGCAATTTTCGTACTGAAACATCTTAATCATGCTGCGGAGGGTTTCTAATG | (Iverson et al., 2016; Mendoza-Suárez et al., 2020) |
| *sfGFP* | ATGCGTAAAGGCGAAGAGCTGTTCACTGGTGTCGTCCCTATTCTGGTGGAACTGGATGGTGATGTCAACGGTCATAAGTTTTCCGTGCGTGGCGAGGGTGAAGGTGACGCAACTAATGGTAAACTGACGCTGAAGTTCATCTGTACTACTGGTAAACTGCCGGTACCTTGGCCGACTCTGGTAACGACGCTGACTTATGGTGTTCAGTGCTTTGCTCGTTATCCGGACCATATGAAGCAGCATGACTTCTTCAAGTCCGCCATGCCGGAAGGCTATGTGCAGGAACGCACGATTTCCTTTAAGGATGACGGCACGTACAAAACGCGTGCGGAAGTGAAATTTGAAGGCGATACCCTGGTAAACCGCATTGAGCTGAAAGGCATTGACTTTAAAGAAGATGGCAATATCCTGGGCCATAAGCTGGAATACAATTTTAACAGCCACAATGTTTACATCACCGCCGATAAACAAAAAAATGGCATTAAAGCGAATTTTAAAATTCGCCACAACGTTGAGGATGGCAGCGTGCAGCTGGCTGATCACTACCAGCAAAACACTCCAATCGGTGATGGTCCTGTTCTGCTGCCAGACAATCACTATCTGAGCACGCAAAGCGTTCTGTCTAAAGATCCGAACGAGAAACGCGATCATATGGTTCTGCTGGAGTTCGTAACCGCAGCGGGCATCACGCATGGTATGGATGAACTGTACAAATGAGCTT | (Geddes et al.,2019) c3m, Addgene CIDAR MoClo Vol.1 Extension (kit #1000000161) |
| *sfYFP* | ATGCGTAAAGGCGAAGAGCTGTTCACTGGTGTCGTCCCTATTCTGGTGGAACTGGATGGTGATGTCAACGGTCATAAGTTTTCCGTGCGTGGCGAGGGTGAAGGTGACGCAACTAATGGTAAACTGACGCTGAAGTTCATCTGTACTACTGGTAAACTGCCGGTACCTTGGCCGACTCTGGTAACGACGCTGACTTATGGTGTTCAGTGCTTTGCTCGTTATCCGGACCATATGAAGCAGCATGACTTCTTCAAGTCCGCCATGCCGGAAGGCTATGTGCAGGAACGCACGATTTCCTTTAAGGATGACGGCACGTACAAAACGCGTGCGGAAGTGAAATTTGAAGGCGATACCCTGGTAAACCGCATTGAGCTGAAAGGCATTGACTTTAAAGAAGACGGCAATATCCTGGGCCATAAGCTGGAATACAATTTTAACAGCCACAATGTTTACATCACCGCCGATAAACAAAAAAATGGCATTAAAGCGAATTTTAAAATTCGCCACAACGTGGAGGATGGCAGCGTGCAGCTGGCTGATCACTACCAGCAAAACACTCCAATCGGTGATGGTCCTGTTCTGCTGCCAGACAATCACTATCTGAGCTACCAAAGCGTTCTGTCTAAAGATCCGAACGAGAAACGCGATCATATGGTTCTGCTGGAGTTCGTAACCGCAGCGGGCATCACGCATGGTATGGATGAACTGTACAAA | (Pédelacq et al., 2006) c51m, Addgene CIDAR MoClo Vol.1 Extension (kit #1000000161) |
| *sfCFP* | ATGCGTAAAGGCGAAGAGCTGTTCACTGGTGTCGTCCCTATTCTGGTGGAACTGGATGGTGATGTCAACGGTCATAAGTTTTCCGTGCGTGGCGAGGGTGAAGGTGACGCAACTAATGGTAAACTGACGCTGAAGTTCATCTGTACTACTGGTAAACTGCCGGTACCTTGGCCGACTCTGGTAACGACGCTGACTTGGGGTGTTCAGTGCTTTGCTCGTTATCCGGACCATATGAAGCAGCATGACTTCTTCAAGTCCGCCATGCCGGAAGGCTATGTGCAGGAACGCACGATTTCCTTTAAGGATGACGGCACGTACAAAACGCGTGCGGAAGTGAAATTTGAAGGCGATACCCTGGTAAACCGCATTGAGCTGAAAGGCATTGACTTTAAAGAAGACGGCAATATCCTGGGCCATAAGCTGGAATACAATTACATCAGCGACAATGTTTACATCACCGCCGATAAACAAAAAAATGGCATTAAAGCGAATTTTAAAATTCGCCACAACGTGGAGGATGGCAGCGTGCAGCTGGCTGATCACTACCAGCAAAACACTCCAATCGGTGATGGTCCTGTTCTGCTGCCAGACAATCACTATCTGAGCACGCAAAGCGTTCTGTCTAAAGATCCGAACGAGAAACGCGATCATATGGTTCTGCTGGAGTTCGTAACCGCAGCGGGCATCACGCATGGTATGGATGAACTGTACAAATAAAGGT | (Pédelacq et al., 2006) c91m, Addgene CIDAR MoClo Vol.1 Extension (kit #1000000161) |
| *mScarlet-I* | ATGGTCAGTAAAGGCGAAGCAGTTATCAAAGAGTTCATGCGCTTCAAAGTTCATATGGAAGGGTCGATGAACGGGCACGAATTTGAAATTGAAGGCGAAGGCGAAGGCCGCCCATATGAAGGGACCCAAACCGCAAAGCTTAAGGTTACTAAAGGCGGTCCATTACCCTTTTCGTGGGACATTTTAAGCCCACAGTTTATGTACGGGAGTCGCGCTTTCATCAAGCACCCTGCGGACATCCCAGATTACTACAAACAGTCTTTCCCCGAGGGGTTCAAGTGGGAGCGCGTGATGAACTTCGAGGATGGCGGAGCCGTGACGGTCACCCAAGATACCTCTTTGGAGGACGGTACGTTGATCTACAAAGTGAAATTGCGTGGCACGAATTTTCCACCTGATGGGCCTGTCATGCAGAAAAAGACAATGGGATGGGAAGCTTCCACGGAGCGCCTTTACCCAGAGGACGGTGTTCTTAAAGGGGATATCAAAATGGCGCTGCGTCTTAAAGATGGAGGCCGCTACCTGGCGGACTTCAAGACTACTTACAAGGCCAAAAAACCAGTGCAGATGCCGGGTGCGTACAATGTAGATCGTAAATTAGATATTACAAGTCACAATGAAGATTACACGGTCGTAGAGCAGTATGAGCGCAGTGAGGGGCGTCACTCTACGGGCGGTATGGACGAGTTATACAAGTAAAGGT | (Bindels et al., 2016) c99m, Addgene CIDAR MoClo Vol.1 Extension (kit #1000000161) |
| *AzamiRed1.0* | ATGGTTAGTGTTATTAAGGAAGAAATGAAGATAAAGTTGCGCATGGAAGGTACAGTCAATGGCCACAATTTTGTCATTGAGGGTGAGGGTAAAGGTAACCCGTACGAAGGGACGCAAACAATGGACCTTAAGGTGACGGAAGGTGGGCCTCTCCCTTTCGCTTACGACATACTGAGTCCGCAATTCATGTATGGGTCCAAAGCCTTTATTAAGTATCCAGCAGACATCCCAGATTATTTCAAACAATCATTCCCAGAGGGGTTCCATTGGGAGCGAGTTATGACATACGAGGATGGCGGGGTCTGTACTGCCACACAAAACACTTCACTGCGTGGCGATTGCTTCTTCTATGATGTCCGTTTTGACGGAGTGAATTTCCCGCCGAACGGCCCGGTGATGCAGAAGAAAACTCTCGGTTGGGAGCCTAGTACTGAGAAGATGTACGTGCGTGACGGTGTGCTGAAGGGTGACGTAATTAAGGCACTGCTGTTAGAGGGTGGCGGACACTACCGATGCGACTTTAAAACTACCTACAAGGCTAAGAAAGACGTACGGTTACCAGGTTACCATTTCGTCGACCATCGCATAGAGATATTGAAGCACGATAAAGATTATAATAAGGTGAAGCAATACGAGAACGCTGTGGCCCGTTATTCGATGCTCCCGAGCCAAGCGAAATAAAGGT | (Imamura et al., 2023) |
| *mARs1* | ATGGTGTCCGTTATTAAGGAAGAAATGAAGATAAGACTCCGCATGGAAGGGACAGTAAATGGACACAACTTTGTGATCGAAGGCGAGGGGAAAGGGAATCCATACGAGGGTACACAAACGCTGGACTGCAAGGTAACGGAGGGAGGTCCGCTCCCGTTTGCTTACGACATCCTGTCTCCTCAATTTATGTACGGTTCGAAACCGTTCATTAAATATCCGGCGGACATCCCAGATTATTTCAAACAATCATATCCAGAGGGCCAACACTGGGAGCGGGTCATGACTTACGAGGACGGCGGTGTCTGTACCGCCACCCAAAACTCTAGCCTGAGAGGAGATTGTTTCTTTTATGACGTGCGGTTCGACGGTACGAATTTTCCACCAAACGGCCCGGTGATGCAGAAGAAAACGCTCGGGTGGGTGCCCTCGAGTGAGAAGATGTACGTTCGAGATGGTGTGTTGAAAGGGGATGTATCGAAGGCACTGCTTCTCGAGGGCGGTGGCCACTACCGCTGTGACTTCAAGACGACGTACAAAGCTAAGAAGGACGTAAGACTGCCAGGCGCTCACAAGGTAGATCACCGAATAGAGATCCTGAAGCACGACAAGGATTACAATAACGTTAAGTTATACGAGATTGCAGTCGCCCGCTACAGTTAAAGGT | (Imamura et al., 2023) |
| *mRFP1* | ATGGCTTCCTCCGAAGATGTTATCAAAGAGTTCATGCGTTTCAAAGTTCGTATGGAAGGTTCCGTTAACGGTCACGAGTTCGAAATCGAAGGTGAAGGTGAAGGTCGTCCGTACGAAGGTACCCAGACCGCTAAACTGAAAGTTACCAAAGGTGGTCCGCTGCCGTTCGCTTGGGACATCCTGTCCCCGCAGTTCCAGTACGGTTCCAAAGCTTACGTTAAACACCCGGCTGACATCCCGGACTACCTGAAACTGTCCTTCCCGGAAGGTTTCAAATGGGAACGTGTTATGAACTTCGAGGACGGTGGTGTTGTTACCGTTACCCAGGACTCCTCCCTGCAAGACGGTGAGTTCATCTACAAAGTTAAACTGCGTGGTACCAACTTCCCGTCCGACGGTCCGGTTATGCAGAAAAAAACCATGGGTTGGGAAGCTTCCACCGAACGTATGTACCCGGAAGATGGTGCTCTGAAAGGTGAAATCAAAATGCGTCTGAAACTGAAAGACGGTGGTCACTACGACGCTGAAGTTAAAACCACCTACATGGCTAAAAAACCGGTTCAGCTGCCGGGTGCTTACAAAACCGACATCAAACTGGACATCACCTCCCACAACGAGGACTACACCATCGTTGAACAGTACGAACGTGCTGAAGGTCGTCACTCCACCGGTGCTTAA | (Campbell et al., 2002) c5m, Addgene CIDAR MoClo Vol.1 Extension (kit #1000000161) |

**Table S3**

The nitrogen-free rooting solution was prepared as previously described (48) with the following composition:

| **Component** | **Concentration** |
| --- | --- |
| CaCl₂·2H₂O | 1 mM |
| KCl | 100 µM |
| MgSO₄·7H₂O | 800 µM |
| Fe-EDTA | 10 µM |
| H₃BO₃ | 35 µM |
| MnCl₂·4H₂O | 9 µM |
| ZnCl₂ | 0.8 µM |
| Na₂MoO₄·2H₂ | 0.5 µM |
| CuSO₄·5H₂O | 0.3 µM |
| KH₂PO₄ | 50 g/L |
| Na₂HPO₄ | 56.8 g/L |

The pH of the solution was adjusted to 7.0 ± 0.2 using NaOH or HCl before use.

**Table S4**The Jensen's media was prepared as previously described (49) with the following composition:

| **Jesnsen’s Media + 0.5 mM nitrogen (10x for 1 L)** | **Weight** |
| --- | --- |
| Calcium phosphate dibasic (CaHPO_4_) | 10 g |
| Potassium phosphate dibasic (K_2_HPO_4_) | 2 g |
| Magnesium sulfate heptahydrate (MgSO_4_: 7H_2_O) | 2 g |
| Ferrous chloride anhydrous (FeCl_2_) | 1 g |
| Potassium Nitrate (KNO_3_) | 505 mg |
| 1000x trace mineral solution | 10 mL |
| **Trace Mineral Solution (1000x per 1 L)** | **Weight** |
| Biotin | 0.4 g |
| CuSO_4_ | 0.5 |
| H_3_BO_3_ | 1 g |
| MnCl_2._ 4H_2_O | 0.5 g |
| Na_2_EDTA | 10 g |
| Na_2_MoO_4_ | 1 g |

The pH of the solution was adjusted to 7.0 ± 0.2 using NaOH or HCl before use.

**Table S5**Composition of ½ Hoagland’s media, modified from Hoagland and Arnon 1950 (50):

**Stock I 400x, per liter: Nitrogen source**

236.2g Ca (NO_3_)_2_ 4H_2_O (calcium nitrate)

101.11g KNO_3_ (potassium nitrate)

**Stock II 1000x, per liter:**

246.48g MgSO_4_ 7H_2_O (magnesium sulfate)

**Stock III 2000x, per liter: Iron source**

36.7g NaFeEDTA (Ethylenediaminetetraacetic acid iron (III) sodium salt)

Wrap the bottle in foil to keep out light.

**Stock IV 5000x, per liter:**

13.6g KH_2_PO_4_ (potassium phosphate, monobasic)

**Stock V 1000x, per liter: Micronutrients**

618.4mg H_3_BO_3_ (boric acid)

48.4mg Na_2_MoO_4_ 2H_2_O (sodium molybdate or molybdic acid)

287.6mg ZnSO_4_ 7H_2_O (zinc sulfate)

395.8mg MnCl_2_ 4H_2_O (manganese chloride)

124.8mg CuSO_4_ 5H_2_O (cupric sulfate)

47.6mg CoCl_2_ 6H_2_O (cobalt chloride)

1.25ml 10M HCl or 1.033ml 12.1M HCl

**Stock VI 1000x, per liter: Buffer**

97.5g MES or 106.6g MES monohydrate.

**½ Hoagland media without nitrogen source, 10 liters working:**

Nil Stock I

10ml Stock II

5ml Stock III

2ml Stock IV

10ml Stock V

10ml Stock VI

Adjust the volume to 10 liters and the pH to 6.1.

**Table S6**

*Cytation^TM^ 5* image capture settings for pea nodules.

| **Parameters** | **Fluorescent Protein** | **P*nifH* imaging** | **P*uni* imaging** |
| --- | --- | --- | --- |
| **LED Intensity** | BF / YFP / RFP / CFP | 3 / 10 / 10 / 10 | 3 / 10 / 10 / 10 |
| **Integration Time (ms)** | BF | 100 | 100 |
|  | YFP | 5 | 40 |
|  | mRFP1 / AzamiRed1.0 / mARs1 / mScarlet-I | 116 / 157 / 24 / 24 | 72 / 157 / 30 / 24 |
|  | CFP | 5 | 12 |
| **Gain** | BF | 0.8 | 0 |
|  | YFP | 19.5 | 20 |
|  | mRFP1 / AzamiRed1.0 / mARs1 / mScarlet-I | 24 / 24 / 24 / 24 | 27 / 24 / 30 / 24 |
|  | CFP | 24 | 20 |

**Table S7**

*Cytation^TM^ 5* image capture settings for lotus nodules.

| **Parameters** | **Fluorescent Protein** | **P*nifH* imaging** | **P*uni* imaging** |
| --- | --- | --- | --- |
| **LED Intensity** | BF / YFP / RFP / CFP | 9 / 10 / 2 / 3 | 9 / 10 / 10 / 10 |
| **Integration Time (ms)** | BF / YFP / RFP / CFP | 5 / 5 / 5 / 5 | 5 / 30 / 45 / 17 |
| **Gain** | BF / YFP / RFP / CFP | 0.8 / 3.17 / 1 / 1 | 0.8 / 20 / 24 / 24 |
